# Strain-specific transcriptional responses overshadow salinity effects in a marine diatom sampled along the Baltic Sea salinity cline

**DOI:** 10.1038/s41396-022-01230-x

**Published:** 2022-04-05

**Authors:** Eveline Pinseel, Teofil Nakov, Koen Van den Berge, Kala M. Downey, Kathryn J. Judy, Olga Kourtchenko, Anke Kremp, Elizabeth C. Ruck, Conny Sjöqvist, Mats Töpel, Anna Godhe, Andrew J. Alverson

**Affiliations:** 1 Department of Biological Sciences, University of Arkansas, Fayetteville, AR USA; 2 Department of Statistics, University of California, Berkeley, CA USA; 3 Department of Applied Mathematics, Computer Science and Statistics, Ghent University, Ghent, Belgium; 4 Bioinformatics Institute Ghent, Ghent University, Ghent, Belgium; 5 Department of Marine Sciences, University of Gothenburg, Gothenburg, Sweden; 6 Leibniz-Institute for Baltic Sea Research Warnemünde, Rostock, Germany; 7 Environmental and Marine Biology, Åbo Akademi University, Åbo, Finland

**Keywords:** Water microbiology, Microbial ecology, Molecular ecology, Transcriptomics

## Abstract

The salinity gradient separating marine and freshwater environments represents a major ecological divide for microbiota, yet the mechanisms by which marine microbes have adapted to and ultimately diversified in freshwater environments are poorly understood. Here, we take advantage of a natural evolutionary experiment: the colonization of the brackish Baltic Sea by the ancestrally marine diatom *Skeletonema marinoi*. To understand how diatoms respond to low salinity, we characterized transcriptomic responses of acclimated *S. marinoi* grown in a common garden. Our experiment included eight strains from source populations spanning the Baltic Sea salinity cline. Gene expression analysis revealed that low salinities induced changes in the cellular metabolism of *S. marinoi*, including upregulation of photosynthesis and storage compound biosynthesis, increased nutrient demand, and a complex response to oxidative stress. However, the strain effect overshadowed the salinity effect, as strains differed significantly in their response, both regarding the strength and the strategy (direction of gene expression) of their response. The high degree of intraspecific variation in gene expression observed here highlights an important but often overlooked source of biological variation associated with how diatoms respond to environmental change.

## Introduction

The salinity gradient separating marine and freshwater environments represents one of the major ecological divides structuring microbial diversity [1]. Differences in osmotic pressure impede marine–freshwater transitions, and as a consequence, transitions are generally rare, occur on longer evolutionary timescales [2, 3], and have led to repeated bursts of diversification in freshwater environments [4]. Identifying the processes underlying marine–freshwater habitat transitions is fundamental to our understanding of lineage diversification and habitat structuring on evolutionary timescales [5], as well as short-term adaptive potential to climate change as melting ice caps, altered precipitation patterns, and changes in oceanic currents result in freshening of large regions and local changes in the seasonal or annual cycling of salinity regimes [6, 7]. Permanent establishment of ancestrally marine organisms in freshwaters depends on the ability of individual colonists to survive the initial hypoosmotic stress, acclimate to low salinity, and ultimately adapt to their new environment [8]. Consequently, these transitions should happen gradually [4, 5], and euryhaline or brackish species that can tolerate a wide range of salinities are probably more likely to successfully cross the salinity divide. Studies focused on these taxa can provide key insights into the cellular processes that help mediating marine–freshwater transitions.

**Fig. 1:**
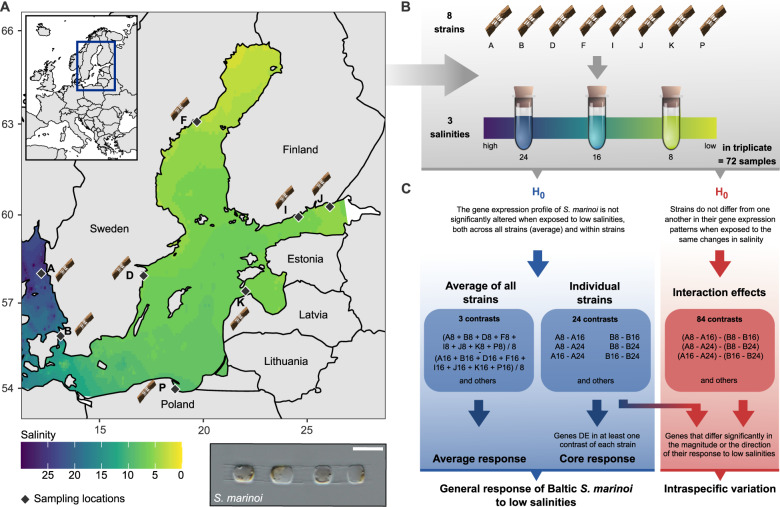
Experimental design. **A** Field sampling. Natural salinity gradient in the Baltic Sea based on salinity measurements from surface samples (0–10 m depth) and interpolated across the Baltic Sea for the period 1990–2020. Salinity measurements were downloaded from ICES (ICES Dataset on Ocean Hydrography, 2020. ICES, Copenhagen) and Sharkweb (https://sharkweb.smhi.se/hamta-data/). Diamonds identify sampling locations for *S. marinoi*. The inset figure on the top left shows the general geographic area in which the Baltic Sea is located. The bottom right figure shows a light micrograph of a *S. marinoi* culture (scale bar = 10 μm). **B** Laboratory experiment. Experimental design of the laboratory experiment carried out in this study. Eight strains of *S. marinoi* were exposed to three salinity treatments (8, 16, and 24) in triplicate, resulting in 72 RNA-seq libraries. **C** Statistical analyses. Overview of the null hypotheses and contrasts tested in this study. Our experimental design allowed characterization of the general response of acclimated *S. marinoi* to low salinities as well as intraspecific variation. The lower blue arrows indicate which data were incorporated in the average and core responses, which together were used to define the general response of *S. marinoi*. Genes with significant interaction effects were subdivided in two categories using logFC values of the individual strains (blue-red gradient arrow), distinguishing genes that differed significantly in either the magnitude or direction of their response to low salinities. The first category includes genes that were DE in one strain but not the others, or that were DE in multiple strains but with significant differences in logFC values in the same direction. Genes of the second category were significantly upregulated in some strains, whereas they were significantly downregulated in other strains.

Here, we take advantage of a natural evolutionary experiment: the colonization of one of the world’s largest brackish water bodies, the Baltic Sea, by the ancestrally marine diatom *Skeletonema marinoi* (Fig. 1A). Geologically, the Baltic Sea is young, with sea ice from the last glacial maximum having fully receded only ~10,000 years ago and inundation of saline waters from the adjacent North Sea occurring ~8000 years ago [9]. Today, freshwater input from rivers and precipitation, combined with inflow of saline bottom-waters from the North Sea through the Danish straits, results in a latitudinal and vertical salinity gradient ranging from near fresh to fully marine conditions [9, 10] (Fig. 1A). The Baltic salinity gradient strongly structures aquatic biodiversity at the species and population levels [11–13], including *S. marinoi* [14], which is the dominant phytoplankton species and one of the main primary producers in the area [15, 16]. Sediment cores showed that *S. marinoi* has been present in the Baltic Sea since the marine inundation or shortly thereafter [17]. Although *S. marinoi* is ancestrally marine [18, 19], it can tolerate a wide range of salinities and is common along the entire salinity gradient, from the North Sea coast to the upper reaches of the Baltic Sea [14]. Previous work showed reduced gene flow between a high-salinity North Sea population and a low-salinity Baltic Sea population, which exhibited lower genetic diversity and optimal growth at lower salinity, consistent with local adaptation [14]. Thus, *S. marinoi* presents an excellent system for understanding how marine diatoms adapt to low salinity environments.

We combined a laboratory common garden experiment with RNA-sequencing (RNA-seq) to characterize the response of *S. marinoi* to low salinity (Fig. 1). We collected eight strains along the Baltic Sea salinity cline, acclimated them to a range of salinities, and compared gene expression between high and low salinity treatments. Natural populations of *S. marinoi* exhibit a broad range of variability in several ecophysiological traits [20–22]. The inclusion of multiple strains in our experiment allowed us to characterize variation in the salinity response as well, including which aspects of the response are shared or different among strains.

## Material and methods

### Sample collection, experimental design, and RNA processing

We collected sediment samples from eight locations across the Baltic Sea (Fig. 1A) and stored them in the dark at 5 °C. We germinated *S. marinoi* resting cells into monoclonal cultures [23] that were kept at their native salinity (Table 1) for 12–26 months prior to the experiment. Strain identity was confirmed by sequencing the LSU rRNA gene (D1–D2 region). During our experiment, we grew the strains in triplicate at three salinities (8, 16, and 24), a design that included both biological (eight strains) and technical replication (three replicates/strain) (Fig. 1B). Strains were regularly reinoculated to maintain exponential growth, and growth rates were monitored via chlorophyll *a* fluorescence. Starting from day 11 cells were harvested for RNA-seq, at which point all strains were acclimated to the experimental salinities. For each strain, two harvests were pooled to obtain sufficient RNA for sequencing. We mapped quality-controlled and trimmed RNA-seq reads against the reference genome of *S. marinoi* strain RO5AC v1.1 with STAR [24], followed by gene-level read quantification with HTSeq [25]. We obtained functional annotations for all genes with InterProScan, KofamKOALA, and BLAST+ searches against Swissprot/Uniprot [26–28]. We detected orthologs of *S. marinoi* genes in other diatom genomes with OrthoFinder [29] and predicted protein targeting with MitoProt, HECTAR, SignalP, ASAFind, and TargetP [30–34]. The Supplementary Methods contain full details on the experimental design and the analyses.

**Table 1:** Details of the *S. marinoi* strains used in this study.

Collection	Culture	Strain	DCG						Isolation	Culture	Original	Culture
ID	ID	ID	accession	GenBank	Country	GPS (N/E)	Collector	Year	date	medium	salinity	salinity
AJA304 (A)	AJA304-05	A.2.21b	DCG 1232	OM112317	Sweden	58.02868/11.13738 (*)	A.Godhe	2014	2017-03-28	L1	15-33	24
AJA305 (B)	AJA305-18	B.2.19b	DCG 1236	OM112318	Sweden	55.97744/12.69058	A.Godhe	2010	2017-04-07	L1	12-15	16
AJA332 (D)	AJA332-09	D.1.27a	DCG 1238	OM112319	Sweden	58.33200/16.70583	A.Godhe,	2017	2018-05-14	WC + salt	8-9	8
							B.Andersson					
AJA328 (F)	AJA328-03	F.1.2a	DCG 1237	OM112320	Sweden	63.65317/18.95200 (*)	A.Godhe	2016/2017	2018-03-15	WC + salt	~8	8
AJA311 (I)	AJA311-04	I.3.11a	DCG 1233	OM112321	Finland	60.18000/25.50700	A.Kremp	2015	2017-03-09	WC + salt	5–6	5
AJA313 (J)	AJA313-31	J.3.42b	DCG 1235	OM112322	Finland	60.38964/27.37518 (*)	A.Kremp	2016	2018-04-22	WC + salt	4-5	8
AJA318 (K)	AJA318-23	K.3.3a	DCG 1234	OM112323	Estonia	57.81670/22.28330	S.Sildever	2016	2018-05-23	WC + salt	~8	8
AJA333 (P)	AJA333-06	P.2.6a	DCG 1239	OM112324	Poland	54.44778/18.57611	A.Witkowski	2018	2018-05-16	WC + salt	5-7	5

The column labelled *Year* indicates the collection year of the sediment samples (see *Collection ID*: letters between brackets refer to sampling localities in Fig. 1) from which *S. marinoi* strains (see *Culture ID* and *Strain ID*) were germinated. All strains are publicly available from the BCCM/DCG diatom culture collection (https://bccm.belspo.be/about-us/bccm-dcg) under the DCG accession numbers listed in the table. GenBank accession numbers refer to LSU D1–D2 rRNA gene sequences used for strain identification. The salinity values indicate the salinity of the natural sample from which the respective strains were isolated (*Original salinity*) and in which they were maintained prior to the experiment (*Culture salinity*). The culture medium indicated in the table represents the medium in which the strains were maintained prior to the experiment. Throughout the experiment, artificial sea water (ASW medium, see Supplementary Information for the recipe) was used to ensure equal nutrient levels in all salinities. GPS coordinates indicated with an asterisk (*) represent approximate sampling locations.

### Hypothesis testing and GO enrichment

We tested two sets of null hypotheses, using edgeR [35] and stageR [36] (Fig. 1C). The first set tested whether gene expression was different across the salinity gradient for each strain separately and for all strains together, using 27 contrasts (Fig. 1C). Compared to solely testing the average salinity effect, simultaneously accounting for the individual strains increases the power to find differentially expressed (DE) genes, as the strain effect incorporates variability that would otherwise be unaccounted for. The second set of hypotheses tested for an interaction effect between strain and salinity, i.e., whether there are strain-specific responses to changes in salinity. Here, we defined 84 contrasts, testing each pairwise combination of strains within all three salinity combinations (Fig. 1C). We tested the two sets of hypotheses separately using stageR’s stage-wise testing procedure, thus controlling the gene-level false discovery rate (FDR) within each set at 5% [36, 37].

Gene ontology (GO) enrichment was done with TopGO (Overrepresentation analysis, ORA) [38] and CAMERA (gene set enrichment analysis, GSEA) [39]. For ORA, we performed separate GO enrichment for genes that are up- or downregulated in low salinities in each strain or the average response. For GSEA, we performed GO enrichment on each contrast of the individual strains and average effects. Redundant GO terms were removed with REVIGO [40]. Further details are outlined in the Supplementary Methods.

## Results

### Response of Baltic *S. marinoi* to low salinity

**Fig. 2:**
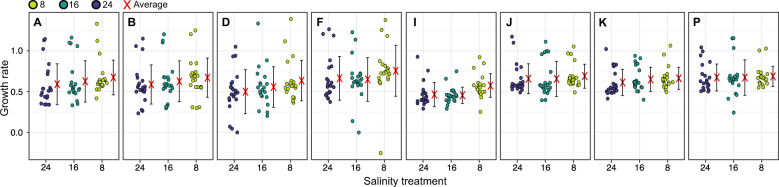
Growth response of Baltic *S. marinoi* in low salinities. Growth rates of the eight *S. marinoi* strains examined in this study at three different salinities. Each point represents a single estimate of the slope of the natural logarithm of in vivo relative fluorescence against time for each sequential transfer, using a horizontal jitter of points to avoid overplotting. The red crosses and vertical black lines represent the average and standard deviation across all replicates and sequential transfers, respectively.

All strains grew well across the salinity range in our study (Fig. 2). The growth rates are within the range observed in previous work [14]. However, salinity reaction norms showed the same pattern for each strain, with slightly higher (but not significant) growth rates at lower salinities (Fig. 2). This contrasts previous work that found lower growth optima in *S. marinoi* strains from low salinity environments compared to those from high salinities [14].

**Fig. 3:**
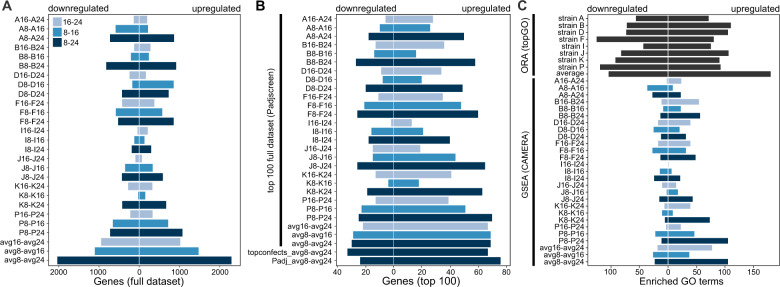
Transcriptome response of Baltic *S. marinoi* to low salinities. **A** Number of DE genes at a 5% FDR-level in the average response and the individual strains. The number of DE genes is indicated separately for each contrast, distinguishing between genes that are up or downregulated. **B** Direction of DE in the top 100 genes of the average response and individual strains as selected by *p* value or logFC. For each contrast in the average and individual strains (vertical black bar), the direction of DE is indicated for the top-100 genes selected by stageR’s FDR-adjusted *p* value of the global null hypothesis (Padjscreen). Thus, although a gene can have a high *p* value on a dataset-wide level, it is not necessarily DE in each individual contrast. In addition, we show the top-100 genes selected by logFC (topconfects [100]) and the contrast-specific 5 % FDR-controlled *p* value (Padj) for the 8–24 contrast of the average effects, as this contrast showed the greatest number of DE genes in (**A**). **C** Number of enriched GO terms for the ORA and GSEA analyses. The number of up- and downregulated GSEA GO terms represents the output classification by CAMERA. The number of enriched GO terms includes Biological Process, Molecular Function, and Cellular Component GO terms, prior to removal of redundant GO terms by REVIGO.

RNA-seq reads of all strains mapped equally well to the reference genome. In the combined average- and strain-specific response, 7905 of the 22,440 predicted genes in the *S. marinoi* genome were DE using a 5% FDR (Supplementary Fig. 1). Of the DE genes, 1652 received no functional annotation. The number of DE genes in the three contrasts of the average response (5343) was greater than that of any individual strain (Fig. 3A, Supplementary Figs. 1–5), which is the result of combining data across all eight strains (8 × 3 replicates/salinity). Consequently, the average response allowed us to detect more DE genes, including those with small effect sizes, and shows the benefit of including biological replicates on top of the standard three technical replicates used in many transcriptome studies. For example, whereas the total number of DE genes within individual strains is comparable with a strain of the euryhaline diatom *Thalassiosira weissflogii* under changing salinity, it is much larger in the average response [41].

The 8–24 contrasts consistently showed the most DE genes, and the least generally were found in the 16–24 contrast (Fig. 3A, Supplementary Figs. 1–3). Thus, the largest difference in salinity (8–24), and the lowest salinity (8–16), elicited the greatest transcriptomic responses. The number of up- and downregulated genes was comparable within contrasts (Fig. 3A, Supplementary Figs. 1–3). However, when only considering the top-100 genes based on *p* value or logFC for each contrast, substantially more genes were upregulated in low salinities (Fig. 3B). This indicates that genes with the strongest evidence for DE or the largest effect sizes were more likely to be upregulated in low salinities. Similarly, CAMERA GO enrichment found substantially more enriched GO terms that were upregulated in low salinities (Fig. 3C). Different numbers of significantly up- and downregulated genes between low and high salinities were also detected in *T. weissflogii* [41].

Next, we report on specific genes and pathways that are DE in low salinities. Unless otherwise noted, we focus on the 8–24 contrast of the average response because it represents the strongest response to salinity in our dataset and GO enrichment showed that despite presence of uniquely DE genes in each salinity contrast (Supplementary Figs. 2 and 3), most of the same processes are enriched in the three salinity contrasts of the average response.

#### Metabolic changes in low salinities

**Fig. 4:**
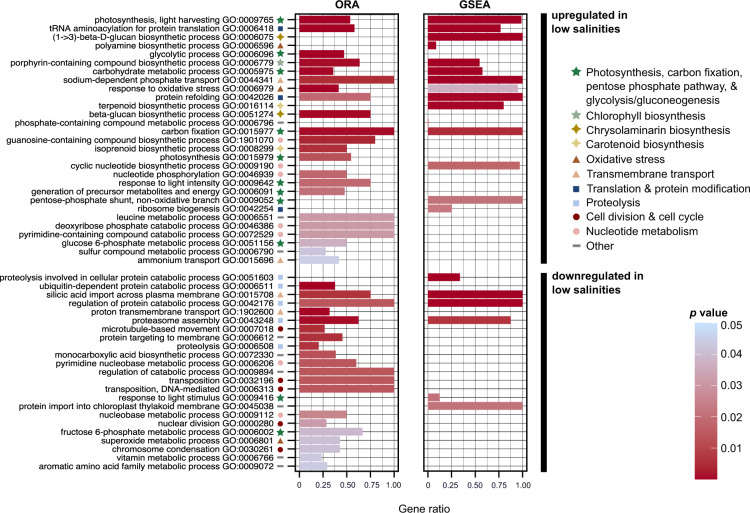
GO enrichment on the average response of *S. marinoi* to low salinities: Biological Process. The results of two types of GO enrichment analyses are shown: ORA (in topGO, Fisher’s exact test, *elim* algorithm) and GSEA (in CAMERA), after removal of redundant terms by REVIGO. For ORA, we classified the total set of DE genes in the average response into two categories, distinguishing between genes that are up- or downregulated in low salinities, regardless of salinity contrast (see Supplementary Methods for details). For CAMERA, we performed GSEA analyses on each individual contrast separately, showing only the 8–24 contrast in this figure. Barplot height indicates the proportion of genes that are DE with a given GO term to the total number of genes with this GO term in the genome of *S. marinoi*. The barplots are colored according to *p* value. Within the set of up- and downregulated genes, the GO terms are ranked from lowest to highest *p* value, using the lowest of two *p* values from ORA or GSEA. Symbols indicate major categories of cellular processes to which a GO term belongs. Only Biological Process GO terms are shown.

In low salinities, *S. marinoi* experienced significant (i) upregulation of genes involved in photosynthesis, Calvin cycle, chlorophyll biosynthesis and glycolysis/gluconeogenesis, including phosphoenolpyruvate carboxylase (PEPC), and (ii) downregulation of genes involved in protein ubiquitination, proteolysis, and aerobic respiration (Fig. 4, Supplementary Figs. 6–9). Most genes involved in the mitochondrial electron transport chain and the TCA cycle, including transcription factor *bZIP14* which regulates the TCA cycle [42], were slightly downregulated (Supplementary Fig. 9A, B). Biosynthesis of fatty acids and the polysaccharide chrysolaminarin (β-1,3/β-1,6-glucan) was upregulated, whereas fatty acid degradation was downregulated (Fig. 4, Supplementary Figs. 6 and 10A).

Genes involved in tRNA-aminoacylation, translational elongation factors, ribosomal proteins, and protein refolding were upregulated, and many genes associated with cell division were downregulated (Fig. 4, Supplementary Figs. 6 and 11). Two genes coding for a sulfolipid biosynthesis protein and a glycosyltransferase (involved in thylakoid membranes and membrane stability, respectively) were upregulated, suggestive of changes in membrane composition. Several transcription factors were also upregulated, including a putative heat stress transcription factor involved in DNA binding of heat shock promoter elements. Two genes coding for an extracellular subtilisin-like serine protease were upregulated, as also observed in diatoms in response to copper deficiency [43]. Finally, although activation of transposable elements has been linked to the diatom stress response, including *S. marinoi* [44–46], most genes involved in transposon activity (transposase, retrovirus-related Pol poly-protein) were downregulated or not DE (Fig. 4, Supplementary Fig. 6).

#### Response to oxidative stress

Multiple mechanisms to deal with reactive oxygen species (ROS) were upregulated in low salinities. This response included genes involved in the xanthophyll cycle, glutathione metabolism, ascorbate peroxidases, catalases, peroxiredoxin, and polyamine biosynthesis from ornithine via ornithine decarboxylase (v Figs 8B, 9C, D, 10B). Carotenoids for the xanthophyll cycle were likely produced primarily through the non-mevalonate pathway (Supplementary Fig. 8B). Several other genes involved in ROS elimination, such as the gene coding for superoxide dismutase (SOD), were either downregulated or not DE (Supplementary Fig. 10B).

#### Transmembrane transport and nitrogen metabolism

Transmembrane transporters for amino acids, polyamines, pyruvate, and essential nutrients such as nitrogen, phosphorus, molybdate, and sulfate were upregulated in low salinities (Supplementary Figs 9D, 12). *Nrt* nitrite/nitrate transporters were highly upregulated, and to a lesser extent also transporters for urea and ammonia. Most of the imported nitrogen is probably directed to the chloroplast, where nitrogen assimilation through ferredoxin-nitrite reductase and GSII-GOGAT_(Fd)_ [47] was upregulated (Supplementary Fig. 9D). In parallel, the anabolic part of the urea cycle was upregulated, including carbamoyl phosphate synthase (Supplementary Fig 9C, D), suggestive of increased recycling of ammonia and biosynthesis of arginino-succinate or arginine. In contrast, silicic acid transporters were downregulated, and this response was most evident in the 16–24 salinity contrast (Supplementary Fig. 12).

#### Osmotic stress response

Our data suggest that *S. marinoi* responded to differences in osmotic pressure by adjusting intracellular osmolyte concentrations to hypoosmotic conditions. This response was larger in the 16–24 contrast compared to the 8–16 contrast (Supplementary Fig. 10B). Although the dimethylsulfoniopropionate (DMSP) pathway remains poorly characterized in diatoms, *S. marinoi*’s homolog of *TpMMT*, a methyltransferase that catalyzes a key reaction in DMSP biosynthesis [48], was strongly downregulated (Supplementary Fig. 10B). In addition, breakdown of the osmolyte taurine via taurine dioxygenase was upregulated (Supplementary Fig. 10B). The magnitude of DMSP downregulation in *S. marinoi* was similar to that of another euryhaline diatom, *Cyclotella cryptica*, grown in comparable salinities, whereas the effect sizes for taurine were larger than reported for *C. cryptica* [49]. Putative *BADH* and *CDH* genes involved in the biosynthesis of the osmolyte glycine betaine from choline [50] were not DE. A putative homolog of the *Thalassiosira pseudonana* gene *TpGSDMT*, involved in the biosynthesis of glycine betaine from glycine [50], was significantly downregulated (Supplementary Fig. 10B). Genes involved in proline metabolism [51] showed inconsistent expression patterns, being up- or downregulated, or not DE (Supplementary Fig. 9D).

**Fig. 5:**
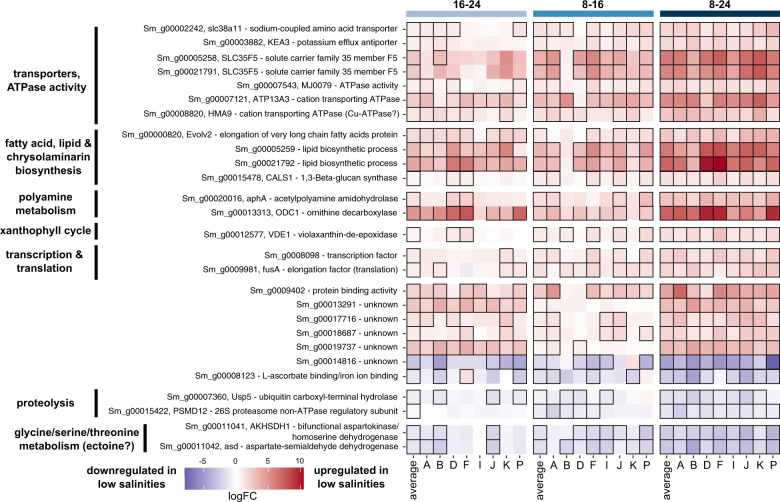
Set of genes that are DE in at least one contrast of each strain: the core response. The heatmap shows logFC values for the individual strains and average response of the 27 core-response genes. The three salinity combinations are indicated on top of the figure. Contrasts that were significant are outlined in black. Row names specify gene names and functional annotations based on Swissprot/Uniprot and/or GO terms. When DE, all genes are consistently up- or downregulated in low salinities in each strain, except for gene *Sm_g00008123*. In the 8–16 contrast, genes *Sm_g00007543* and *Sm_g00005259* are not DE for strains I and K, but appear DE in the figure due to colored edge lines from neighboring squares. Similarly for the 16–24 contrast, *Sm_g00005259* is not DE for strains A and F.

Responses to osmotic stress also included shifts in cation import and export, such as sodium and potassium [49, 52]. Here, most potassium and sodium channels were either upregulated or not DE, and two detected aquaporins had opposite expression patterns (Supplementary Fig. 12). Several transporters for potassium or unknown cations/solutes were DE in all strains, often with large effect sizes (Fig. 5).

### Strain-specific data reveal intraspecific variation and a conserved core response to low salinity

**Fig. 6:**
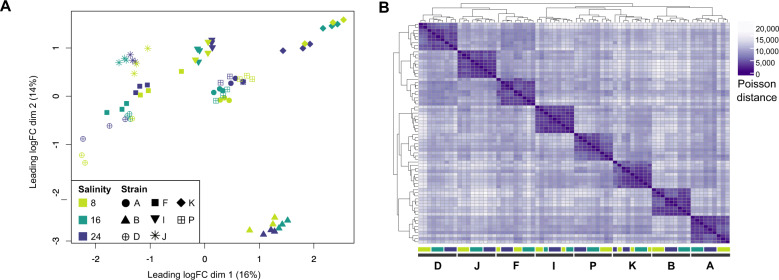
Intraspecific variation in the response of Baltic *S. marinoi* to low salinities. **A** Multidimensional scaling (MDS) plot, showing that samples cluster primarily by strain rather than salinity. Distances between the samples are based on logFC changes in the top-500 genes, selecting the top-500 genes separately for each pairwise comparison between the samples. **B** Poisson-distance heatmap of the full dataset. Colored bars below the heatmap indicate the position of samples belonging to different strains and salinities (Fig. 1A), showing that samples of different strains cluster together.

All previous results were based on the average response (Fig. 1C). However, when we take the responses of individual strains into account, it becomes clear that the strain effect in our dataset exceeded the salinity effect. Strains differed substantially in their responses to low salinities, which was evidenced by a multidimensional scaling plot and poisson-distance heatmap in which samples clustered primarily by strain rather than salinity (Fig. 6). In fact, when combining data of all three salinity contrasts, 1791 genes were uniquely DE in only one strain (and not DE in the average response), and 1628 genes were uniquely DE in one strain and the average response. The number of uniquely DE genes per strain ranged from 103 to 317 genes, and 951 genes were DE only in the average response (Supplementary Fig. 4A). A similar pattern emerged when examining each salinity contrast separately (Supplementary Figure 5). The high number of genes that are DE only in the average response or in one strain plus the average response is due to the higher statistical power provided by combining data of all strains together in the average response.

We defined a core response to low salinities by selecting genes that are DE in at least one contrast of each strain, which resulted in a set of 27 shared genes that are DE in each of the eight strains (Figs. 1C and 5). Obtaining this set of shared genes required subsetting the full set of DE genes, so the 5% FDR could not be guaranteed for these 27 genes. However, these core-response genes were characterized by a combination of high logFC and low *p* values (Fig. 5, Supplementary Fig. 1), thus providing strong evidence for DE in each strain. These genes are also among the top DE genes: 13 overlapped with the top-25 DE genes as ranked by stageR’s FDR-adjusted *p* value of the global null hypothesis (Padjscreen), 22 were part of the top 100, and all were detected within the top-225 genes. Core-response genes upregulated in low salinities were involved in key processes previously identified in the average response, including transport of amino acids and cations, biosynthesis of fatty acids, lipids and chrysolaminarin, polyamine metabolism/biosynthesis, the xanthophyll cycle, and transcription/translation (Fig. 5). By contrast, core-response genes that were downregulated in low salinities were involved in proteolysis or the glycine/threonine/serine pathway (Fig. 5, Supplementary Fig. 13). Seven core-response genes had unknown functions (Fig. 5).

### Interaction effects reveal differences among strains in their response to low salinity

A total of 3857 genes showed significantly different expression patterns between strains with a 5% FDR (interaction effects, Fig. 1C). Of these, 2820 differed between strains in the magnitude of their response to low salinities, whereas far fewer (1037) differed in the direction of their response. However, 92 of the top-100 genes with interaction effects (ranked by stageR’s Padjscreen) differed in the direction of their response (Supplementary Fig. 14). Thus, although more genes showed differences in the magnitude of DE, those with differences in direction of DE dominated the top interaction-effect genes.

**Fig. 7:**
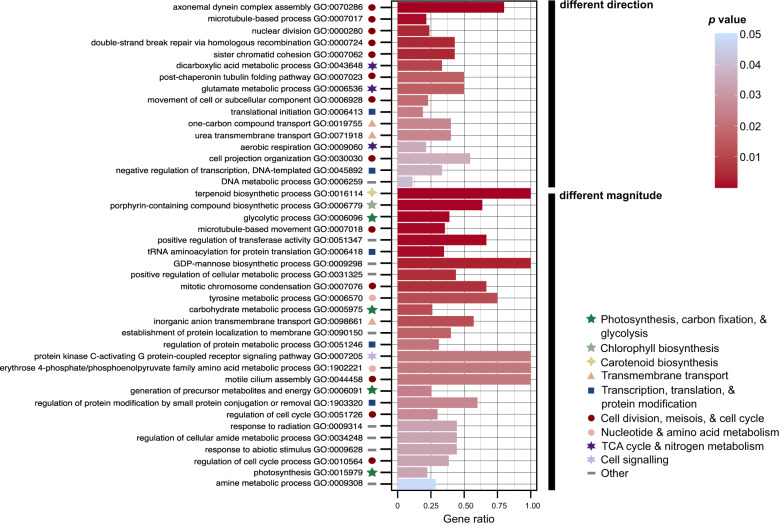
GO enrichment of the interaction effects: Biological Process. The barplot visualizes the significant GO terms retrieved by ORA (topGO, Fisher’s exact test, *elim* algorithm) after removal of redundant GO terms by REVIGO. Two sets of GO enrichment were carried out which distinguished between genes that differ significantly between strains in the direction or magnitude of their response to low salinities. Barplot height indicates the proportion of genes that are DE with a given GO term to the total number of genes with this GO term in the genome of *S. marinoi*. The barplots are colored, and the GO terms ranked, according to *p* value. Symbols indicate major categories of cellular processes to which a GO term belongs. Only Biological Process GO terms are shown.

The two classes of interaction-effect genes were enriched for different processes (Fig. 7, Supplementary Fig. 15). Genes that differed significantly across strains in magnitude were enriched for many of the processes identified in the average response, including photosynthesis, glycolysis, and the biosynthesis of chlorophyll, carotenoids, and fatty acids. By contrast, the gene set that differed significantly between strains in the direction of their response was enriched for transcription regulation, peroxidase activity, aerobic respiration, and urea transmembrane transport (Supplementary Fig. 15). It also contained genes involved in inositol metabolism, cell wall and calcium-binding messenger proteins, and heat shock proteins/chaperones (Supplementary Fig 14). Both classes were enriched for genes involved in translation, cell-cycle progression, mitosis, and meiosis. For example, two genes coding for meiotic recombination protein SPO11-2 were part of the top-100 interaction effects (Supplementary Fig. 14). Nevertheless, depending on the strain and the salinity contrast, their effect sizes were 3–50 times smaller than reported during sexual reproduction in *S. marinoi* [53].

## Discussion

### The average and core response of *S. marinoi* to low salinities

Taken together, our data show that exposure to low salinities triggers a stronger response compared to high salinities (Fig. 3B, C), and suggest that the ancestrally marine diatom *S. marinoi* reprograms its metabolism by upregulating several pathways to function in low salinities. Here, analysis of the average and core responses in *S. marinoi* suggested that in low salinities photosynthesis and carbon fixation are upregulated, and there is less protein recycling. This contrasts with carbon fixation in the euryhaline diatom *T. weissflogii* which was not impacted by low salinities [41]. However, like in *T. weissflogii*, we observed upregulation of PEPC in low salinities [41]. PEPC has multiple functions, including supplying oxaloacetate to the TCA cycle, which is, however, slightly downregulated in *S. marinoi*. In some diatoms, PEPC appears to be involved in the carbon concentrating mechanisms (CCMs) of a C4 mechanism similar to that of plants [54, 55]. Our expression data suggest PEPC might play a similar role in *S. marinoi*. Upregulation of this gene could reflect an increased need to dissipate energy and/or increase CO_2_ concentrations near Rubisco to compensate for a potential decrease in the availability of dissolved inorganic carbon and/or Na^+^-dependent HCO_3_^−^ transport in low salinities, as was suggested for *T. weissflogii* [41]. Alternatively, given that the Calvin cycle is also upregulated in low salinities, upregulation of PEPC might contribute to a net increase of carbon fixation in low salinities.

Glycolysis/gluconeogenesis is upregulated in low salinities. Protein targeting suggests both pathways are (partially) compartmentalized across the chloroplasts, cytosol, and mitochondria (Supplementary Fig. 7E, F), presumably allowing them to run simultaneously to supply precursors for biosynthesis of both fatty acids and polysaccharides [56]. Indeed, genes involved in biosynthesis of fatty acids and chrysolaminarin, an important storage polysaccharide in diatoms [57], were upregulated, including four of the core-response genes. In addition, a BASS2-like pyruvate transporter was upregulated, suggestive of increased transport of pyruvate to the chloroplast where it serves as precursor for fatty acid biosynthesis [58]. Diatoms are known to accumulate storage compounds in unfavorable growth conditions, and to modify the fatty acid and lipid composition of their membranes in response to osmotic changes, which alters membrane permeability and fluidity under salinity stress [59–61]. Upregulation of these genes thus suggests that low salinities represent suboptimal growth conditions for *S. marinoi*.

The hypothesis that low salinities are suboptimal is further supported by expression data that suggest a decrease in nuclear division and silicic acid uptake in low salinities, consistent with a decrease in cell division. Growth rates in the euryhaline diatom *T. weissflogii* also decreased in lower salinity [62], and in the marine diatom *Chaetoceros gracilis*, low salinity was found to negatively affect silicon metabolism [63]. Paradoxically, decreased mitosis was not reflected in our growth data measured from relative chlorophyll *a* fluorescence, which showed approximately equal growth rates across salinities for all strains, with even slightly higher (but not significant) rates in low salinities (Fig. 2). However, upregulation of the chlorophyll-biosynthesis pathway in low salinities, despite constant light levels, suggests that a decreased growth rate could have been masked by an increase in per-cell chlorophyll content. Such increase in chlorophyll content under moderate hypersalinity stress was previously detected in green algae and is thought to drive elevated photosynthesis [64, 65]. Our data suggest that *S. marinoi* adopts a similar response to low salinity. Given that major salinity stress in algae usually results in a decreased chlorophyll content [66] and less photosynthesis [67], our data indicate that although low salinities are not optimal for *S. marinoi*, when acclimated the diatom is not severely stressed in these conditions. Furthermore, this observation might have important consequences for similar experiments that use chlorophyll *a* as a proxy for growth. Further research is necessary to unravel the link between fluorescence, chlorophyll content, and salinity in *S. marinoi*, given that many factors can influence fluorescence measurements [68].

The response of *S. marinoi* to potential oxidative stress experienced in low salinities is complex. On the one hand, our data suggest that proteins are repaired at higher rates in low salinities, which might reflect an increase in damage caused by oxidative stress [69]. In addition, both the xanthophyll cycle and polyamine biosynthesis were strongly upregulated in low salinities. The former plays a critical role in protection from oxidative stress due to excess light, but also from ROS generated by other stressors, such as salinity [70]. Polyamines function in abiotic stress responses in land plants, including salinity stress, by increasing antioxidant enzyme activity, triggering the stress signal transduction chain, and serving an osmolyte function [71]. In diatoms, polyamines are known to increase in response to both heat and salinity stress [72, 73], and our data suggest a similar role in salinity acclimation. Violaxanthin-de-epoxidase (xanthophyll cycle), and two genes involved in polyamine biosynthesis belonged to the core response, underscoring the highly conserved nature of this response. On the other hand, several other genes involved in ROS elimination were slightly downregulated, or not DE, in low salinities, including the SOD gene which is a first line of defense against ROS in land plants and macroalgae [74, 75]. This might indicate that *S. marinoi* was not acutely stressed, but instead reached an adaptive state of long-term ROS management allowing for survival and growth in suboptimal conditions.

Given the approximately equal growth rates across salinities and transcriptomic evidence consistent with decreased cell division in low salinities, the observation of possibly increased nutrient transport and nitrogen assimilation in low salinities suggests a higher per-cell nutrient demand in low salinities. Differences in nutrient uptake and nitrogen assimilation between different salinities have been previously reported in microalgae [76, 77], and could reflect increased biosynthesis of, for example, nitrogen-rich compounds such as polyamines and amino acids, which are essential for the stress response and protein biosynthesis, respectively [59, 78]. Indeed, genes involved in protein biosynthesis are upregulated in low salinities. Previously, higher protein content in lower salinities was observed in the euryhaline diatom *T. weissflogii* [79]. Furthermore, upregulation of amino acid and polyamine transporters pointed to increased demands for compounds essential for cell functioning and/or osmoregulation. This suggests that acclimated *S. marinoi* require more energy and resources to maintain homeostasis in low salinities.

Several of the processes described above were DE in each strain. This core response encompassed 27 genes involved in key processes such as ROS elimination, storage compound biosynthesis, proteolysis, and transmembrane transport. It included one probable transcription factor (*Sm_g00008098*), which is a promising target to unravel the role of gene expression regulation in the salinity response. Increasing the number of technical replicates would likely enlarge the set of core-response genes, as higher replicate numbers improve detection of DE genes, especially those with small effect sizes [80]. Our set of core-response genes is, consequently, not exhaustive but gives a first indication of which genes and processes are likely to be part of a conserved and possibly ancestral response to low salinity in *S. marinoi*.

Finally, it is worth noting that our experiment reflects the salinity-response of *S. marinoi* in optimal growth conditions (e.g., nutrient concentrations). The experimental light levels (30 μmol photons m^−2^ s^−1^) are within the range of the light intensities experienced by natural populations of *S. marinoi* that circulate in the mixed surface layer of the Baltic Sea [81]. We cannot rule out that the salinity response of *S. marinoi* might differ at other light levels or nutrient concentrations [82]. For example, given the importance of increased photosynthesis in low salinities, photoinhibition caused by high light stress could hamper the ability of *S. marinoi* to adequately respond to low salinities.

### Osmoregulation in *S. marinoi*

Diatoms produce a variety of osmolytes, small organic molecules that mitigate hyperosmotic stress typical of marine environments [48–51]. Consequently, a decrease in salinity should trigger a drop in osmolyte biosynthesis. Indeed, the expression pattern in low salinities is consistent with a decrease in biosynthesis of DMSP, taurine, and possibly glycine betaine. Two core-response genes that were strongly downregulated in all strains could be involved in the biosynthesis of another osmolyte, ectoine. These genes encode a bifunctional aspartokinase/homoserine dehydrogenase (*Sm_g00011041*) and an aspartate-semialdehyde dehydrogenase (*Sm_g00011042*) (Fig. 5, Supplementary Fig. 13). Both are involved in the early steps of the glycine/threonine/serine pathway and convert aspartate into aspartate-semialdehyde and/or homoserine. The *S. marinoi* genome contains several other homologs of both genes. When DE, these homologs show opposite expression patterns to the aforementioned genes: they are upregulated in low salinities, following the expression pattern of other genes in this pathway (Supplementary Fig. 13). Peptide-targeting predictions revealed that this pathway is compartmentalized across the chloroplasts, cytoplasm, and mitochondria, presumably allowing *S. marinoi* to run opposite reactions simultaneously while avoiding futile cycles (Supplementary Fig. 13). Given their expression patterns and compartmentalization, *Sm_g00011041* and *Sm_g00011042* are likely not involved in conventional amino acid biosynthesis. Instead, one of their products, aspartate-semialdehyde, is a known precursor for ectoine, an osmolyte common in bacteria [83]. Elevated levels of aspartate-semialdehyde dehydrogenase have been detected in bacteria occupying high salinities [84]. Recently, marine diatoms were found to both biosynthesize ectoine and import ectoine of bacterial origin [85]. Several *S. marinoi* genes may be homologous to bacterial ectoine genes (*ectA*, *ectB*, *ectC*) that convert aspartate-semialdehyde to ectoine. However, low sequence similarity (maximum 47.8%), and lack of downregulation in low salinities, raises doubt about whether those genes are responsible for ectoine biosynthesis in *S. marinoi*. Furthermore, all putative homologs received annotations different from ectoine-related genes in Swissprot/Uniprot. It is possible that diatoms have other unknown genes involved in ectoine biosynthesis, or alternatively, diatoms might provide ectoine precursors (e.g., aspartate-semialdehyde) to extracellular bacteria that synthesize and return ectoine to the diatom. Such metabolite exchanges have been shown to occur in diatom–bacteria interactions [86]. Our expression data are consistent with both scenarios and suggest ectoine might be an important osmolyte in *S. marinoi*.

### Incorporating multiple strains reveals intraspecific variation

The above observations were based on the average and core response of all eight strains, which revealed the general response of Baltic *S. marinoi* to low salinities. However, when taking data of individual strains into account, we observed substantial intraspecific variation in gene expression. Many of the processes identified in the average response differed significantly among strains in magnitude, indicating that strains vary in the strength of their salinity response. This becomes clearer when examining differential expression in different pathways of individual strains (Supplementary Fig. 7–13). Whereas many pathways are almost entirely DE in the average response, this is often not the case for individual strains, which have fewer DE genes than the average response, and differ from one another in which genes are DE as well as the strength of DE (measured as logFC). For example, whereas four strains significantly upregulated almost the entire chlorophyll-biosynthesis pathway, none or only a few genes in this pathway are DE in the other strains (Supplementary Fig. 8A). Similarly, four and five strains significantly upregulated most genes involved in chrysolaminarin and fatty acid biosynthesis, respectively, whereas the other strains have only few genes DE in the same pathways (Supplementary Fig. 10A).

A second set of genes differed among strains in the direction of their response. Thus, strains deviated in their strategies to cope with low salinity. This included both cell wall and calcium-binding messenger proteins as well as heat shock proteins/chaperones. The latter is known to help mitigate elevated salt stress in sea-ice diatoms [87], and our data suggest they play a role also in acclimation to low salinity, although this role is variable across strains. Altogether, these data highlight intraspecific differences in how salinity stress affects cell functioning, including cell-signaling pathways. For example, Ca^2+^-signaling is involved in osmotic sensing in diatoms [88], suggesting strains differ in how they respond to osmotic stress.

The interaction effects included several DE genes related to the cell cycle. Diatoms have an unusual cell cycle that involves progressive cell size reduction through mitotic cell divisions until cell size drops below a species-specific sexual size threshold (SST) at which point the diatom can undergo sexual reproduction with a partner cell (allomixis), usually in response to an environmental trigger [89]. In *S. marinoi*, sexual reproduction in cells below the SST can be induced by shifts to higher salinity [90]. Because cultures were shifted to experimental salinities at least 11 days before RNA harvesting, any sexual reproduction that occurred at this time was long finished upon RNA harvesting [53, 90]. However, when below the SST, *S. marinoi* cells can restore their maximum cell size, through an auxospore-like stage that is not contingent upon a salinity shift and might involve autogamy, apomixis, or vegetative cell enlargement [89, 90]. Although the genes involved in this process are unknown, they likely include much of the cell-cycle genes identified as DE in the interaction effects, suggesting salinity impacts size restoration differently in different strains. This could happen through a direct impact on size restoration (cultures in suboptimal salinity might redirect more energy to maintaining homeostasis or growth, and less to size restoration, or vice versa), or through an indirect impact on growth rates (higher or lower growth rates in different salinities might result in a different proportion of cells under the SST, resulting in more, or less, size restoration). To rule out that size restoration alone was responsible for the interaction effects, we removed from our dataset all *S. marinoi* sex-induced genes identified by [53], as well as genes with GO terms related to cell cycle/mitosis/meiosis (6218 genes, 1797 of which are DE in the interaction effects). Upon removal, samples still clustered principally by strain, not salinity (Supplementary Fig. 16), indicating size restoration is not driving the interaction effects. The high growth rates throughout the experiment confirm this (Fig. 2) [91].

Intraspecific variation in the response to low salinity likely allowed *S. marinoi* to colonize and grow throughout the Baltic Sea. Diatoms can harbor high levels of genotypic and phenotypic variation [20–22, 92–96], and *S. marinoi* even shows trait variation between individual cells of the same clone [21]. The variation in gene expression shown here is a natural extension of these observations. Our study design did not allow testing whether the intraspecific variation is related to the natural salinities at which the different strains occur, as this would require sampling of multiple strains within populations. Nevertheless, visual comparison of gene-expression patterns did not show consistent differences across low- (D, F, I, J, K, P) and high-salinity (A, B) populations (Supplementary Figs. 7–13), nor did those populations cluster separately (Fig. 6). This suggests that if signals of local adaptation along the Baltic salinity cline [14] are due in part to differences in gene expression between high- and low-salinity populations, then those differences are subtle. In any case, substantial intraspecific variation in gene expression in *S. marinoi* exists and is likely critical to its survival, acclimation, and adaptation to a dynamic environment such as the Baltic Sea, where in addition to salinity, marked gradients and seasonal fluctuations in nutrients and temperature also occur [22, 97]. The variation in gene expression observed here increases the chance that at least some cells can survive rapidly fluctuating, potentially adverse, conditions in the short term. Assuming some of this variation is heritable, variable gene expression might also enable long-term evolutionary adaptation by providing targets for natural selection [8, 98].

## Conclusion

Our study design, in which transcriptome data from eight strains were combined into a single analysis, allowed for a holistic view of the response of *S. marinoi* to low salinities in the Baltic Sea, the world’s largest brackish water body. Transcriptome studies often include technical replicates of a single strain, but an increasing number of studies [46, 49] show that experiments without biological replicates are unlikely to be generalizable, as different strains can exhibit markedly different patterns of gene expression. Here, inclusion of both technical and biological replicates allowed us to characterize both conserved and variable responses to low salinity.

We found that when *S. marinoi* experiences long-term exposure to low salinities that mimic the natural Baltic Sea salinity gradient, the diatom is not severely stressed but experiences elevated energy and nutrient demands, increases photosynthesis and storage compound biosynthesis, and deploys a complex response to oxidative stress. This response likely allowed the ancestrally marine *S. marinoi* to grow successfully in low salinity environments and become one of the dominant primary producers in the Baltic Sea. Our analyses revealed substantial intraspecific variability in the response of *S. marinoi* to low salinities, highlighting an important source of biological variation in diatoms. Metatranscriptomics offers a powerful approach for identifying community- and species-level responses to other natural gradients in the ocean [99]. Similar studies of the Baltic Sea would provide valuable corroboration of the results from our controlled laboratory experiment. Altogether, our data indicate variable gene expression plays an important role in how diatoms respond and adapt to environmental change.

## Supplementary information

Supplementary Information

## Data Availability

RNA-seq data are available from the Sequence Read Archive (NCBI) under project number PRJNA772794. The *S. marinoi* reference genome (v1.1) used for read mapping, gene-level count data, and the scripts needed to reproduce all analyses and figures are available from Zenodo (10.5281/zenodo.5266588). All *S. marinoi* strains are publicly available from the BCCM/DCG diatom culture collection (https://bccm.belspo.be/about-us/bccm-dcg) under accession numbers DCG 1232–1239. LSU D1–D2 rRNA gene sequences used for strain identification are available from GenBank (OM112317–OM112324).
