# Supplementary material for: Strain-specific transcriptional responses overshadow salinity effects in a marine diatom sampled along the Baltic Sea salinity cline
Source: ISME J. 2022 Apr 5;16(7):1776–87. doi: 10.1038/s41396-022-01230-x (PMC9213524; doi:10.1038/s41396-022-01230-x)
Supplement: Supplementary file 1 — Supplementary Information [file 41396_2022_1230_MOESM1_ESM.pdf]

SUPPLEMENTARY INFORMATION

Strain-specific transcriptional responses overshadow salinity effects in a marine diatom sampled along the Baltic Sea salinity cline

Eveline Pinseel[1*], Teofil Nakov[1], Koen Van den Berge[2,3,4], Kala M. Downey[1], Kathryn J. Judy[1], Olga Kourtchenko[5], Anke Kremp[6], Elizabeth C. Ruck[1], Conny Sjöqvist[7], Mats Töpel[5], Anna Godhe[5] & Andrew J. Alverson[1*]

[1] Department of Biological Sciences, University of Arkansas, Fayetteville, AR, USA
[2] Department of Statistics, University of California, Berkeley, CA, USA
[3] Department of Applied Mathematics, Computer Science and Statistics, Ghent University, Ghent, Belgium
[4] Bioinformatics Institute Ghent, Ghent University, Ghent, Belgium
[5] Department of Marine Sciences, University of Gothenburg, Gothenburg, Sweden
[6] Leibniz-Institute for Baltic Sea Research, Rostock, Germany
[7] Faculty of Science and Engineering, Åbo Akademi University, Turku, Finland

*Corresponding authors: eveline.pinseel@gmail.com, aja@uark.edu

This Supplementary Information file includes the following items:

- Supplementary Methods
- Supplementary Protocol
- Supplementary Figures 1 – 17
- Supplementary References

SUPPLEMENTARY METHODS

Genotyping *S. marinoi* strains

We genotyped all strains obtained from the sediments for D1-D2 LSU rRNA (28S) to ensure they belonged to the species *S. marinoi*. To this end, we harvested cells by centrifugation and flash freezing in liquid nitrogen after which they were stored at -80 °C until DNA extraction. Frozen cells were broken with 1.0 mm glass beads (Biospec Products, OK, USA) by shaking the tubes for 30 seconds at 3 000 OSM in a Minibeadbeater (Biospec Products, OK, USA). We extracted DNA with the DNeasy® Plant Mini Kit (Qiagen, Hilden, Germany), following the manufacturer's instructions. D1-D2 28S (~600 nt) was amplified by PCR using primers D1R and D2C [1]. PCR reactions consisted of 1.0-5.0 µL DNA extract, 6.5 µL of Failsafe Buffer E (Epicentre Technologies, WI, USA), 0.5 µL of each primer (20 µM stocks), and 0.5 units Taq polymerase, using adjustment with ddH_2O to a final volume of 25 µL. We carried out PCR reactions in a T100 Thermal Cycler (Bio-Rad, Hercules, CA, USA) using the following PCR program for all reactions: initial step at 95 °C for 5 min, followed by 36 cycles (95 °C for 50 s, 56 °C for 60 s, and 72 °C for 60 s), and ending with a final elongation step at 72 °C for 5 min. The resulting PCR products were purified by treatment with an Exonuclease I (Exo) and Shrimp Alkaline Phosphatase (SAP) protocol that included addition of 0.25 µL Exo, 1.75 µL ddH_2O, and 1.0 µL SAP per 25 µL PCR product, followed by heating for 30 min at 37 °C and a 15-min termination step at 80 °C. Only forward strands were sequenced at Eurofins Genomics (Louisville, KY, USA). We edited the sequenced chromatograms with Sequencher v5.1 (Gene Codes Corporation, Ann Arbor, MI, USA). For a strain to be included in our study, its D1-D2 28S sequence (567 bp) had to be 100% identical to the corresponding region of the 28S sequence of *S. marinoi* strain RO5AC for which a genome sequence is available. Consequently, all *S. marinoi* strains in this study had identical D1-D2 28S sequences.

Experimental setup, RNA extraction, and sequencing

Prior to the start of the experiment, monoclonal *S. marinoi* cultures that were germinated from resting cells were grown in their native salinity (Table 1) at 12 °C on a 12:12 light:dark light regime (30 µmol photons m^{-2} s^{-1} light intensity). Growth media used for culture maintenance depended on the salinity: strains maintained at salinity 5 or 8 were kept in WC medium (freshwater medium) [2] with added salts, whereas strains maintained in media with salinity 16 or 24 were kept in L1 medium (enriched seawater) [3] with adjusted salinity (Table 1).

The salinity experiment was run in May-June 2019, at which point the investigated strains were between 12 and 26 months old (Table 1). Throughout the experiment, we used ASW medium with constant nutrient levels, but varying salt concentrations (i.e., 8, 16, and 24) depending on the treatment (see Supplementary Protocol for the recipe of our ASW medium). Prior to the experiment, cultures were acclimated to ASW medium at their native salinity for one week using a 12:12 light:dark light regime at 30 µmol photons m^{-2} s^{-1} light intensity. The experiment was carried out on a single shelf of a Percival incubator (Percival Scientific, IA, USA) using the same light conditions as during acclimation to ASW medium. During the experiment, all 72 cultures were grown simultaneously for one month in 5 mL tubes (disposable borosilicate culture tubes (VWR, PA, USA), 12mm x 75mm, cat #47729-570), filled with 4 mL medium, and divided among three racks. All tubes were pseudorandomized daily to avoid potential batch effects, and cultures were transferred repeatedly to ensure they maintained exponential growth throughout the experiment. We measured in vivo relative chlorophyll *a* fluorescence of each tube daily using a Trilogy fluorometer (Turner Designs, CA, USA), allowing us to monitor growth. Prior to fluorescence measurements, the cultures were placed in the dark for 10 min. The daily fluorescence measurements provided multiple data points for each transfer, all of which were taken during the exponential growth phase. These measurements were used to estimate growth rates by

calculating the slope of the linear regression of the natural logarithm of relative fluorescence by day [4]. Every four days, we transferred the cultures into fresh media, with the first reinoculum taking place after seven days. Prior to reinoculation, cultures were homogenized by pipetting up-and-down with a micropipette, after which cultures were transferred to a new tube with a micropipette. For each reinoculum, we used the relative fluorescence data as a guideline to bring the cell densities in all tubes to the same level (at a relative fluorescence value of 8,000). Cultures were reinoculated six times. Repeated reinoculations allowed obtaining (i) sufficient biomass for RNA-seq (see next paragraph), and (ii) repeated estimates for the growth rate of a strain at a specific salinity.

With each reinoculation starting from day 11 (2nd reinoculation), we harvested cells for RNA extraction. To this end, we harvested all left-over biomass in the experimental tube after the culture was reinoculated. Tubes were centrifuged, supernatant was discarded, and the cell pellets were frozen at -80 °C. To obtain sufficient RNA for sequencing, total RNA was extracted from cells harvested from two reinoculations. For each culture, the second and third reinoculations were used for RNA extraction, with exception of two treatments of strain K.3.3a (second replicate of 8ppt and third replicate of 24 ppt) for which the pellets of the fourth and sixth reinoculation were used due to too low RNA yields from the other pellets. We randomized all samples over five batches and extracted RNA with the Qiagen RNeasy plant mini kit. RNA quality was measured using an Agilent TapeStation 2200 (Agilent, CA, USA). Indexed RNA-seq libraries were prepared with the KAPA mRNA HyperPrep library kit using the standard protocol but with half reaction volumes, after which library quality and quantity was assessed with the TapeStation. All libraries were pooled and sequenced together on a single lane of an Illumina HiSeq4000 (2 x 100 paired-end reads) at the University of Chicago Genomics Facility. An average of 9.6 ± 2.4 million reads per sample was sequenced.

Read trimming and mapping of RNA-seq data

We performed quality-control of the raw reads using FastQC v0.11.5 [5], after which we applied Ktrim v1.1.0 [6] for adapter removal and quality-trimming, using default settings (baseline phred score, -p 33; minimum quality score, -q = 20; minimum read size after trimming, -s = 36). We subsequently mapped the reads against the reference genome of *S. marinoi* strain RO5AC v1.1 using STAR v.2.7.3a [7] with default settings, except for intron size. For the latter, we used *-alignIntronMin 4* and *-alignIntronMax 17105*. These values were based on the *S. marinoi* genome assembly used for our analysis. We used the uniquely mapping reads for gene-level read quantification in HTSeq v0.11.3 [8] via *union* mode. The output of HTSeq was imported in R v4.0.2 (R Core Team, 2020) for further statistical analysis.

Functional annotation of *S. marinoi* genes

We obtained functional annotations of the complete set of *S. marinoi* genes in the reference genome v1.1 using various approaches. First, we used BLAST+ v2.6.0 [9] to run sequence similarity blastp searches of all *S. marinoi* proteins annotated in the genome against the Swissprot (download June 2020) and Uniprot databases (download June 2019), and retained the best hit using a maximum e-value limit of 1e-6. Second, we ran InterProScan v5.36-75.0 [10] against the InterPro collection of protein signature databases, including Gene Ontology (GO) resources, Pfam domains, PRINTS, PANTHER, SMART, SignalP_EUK, and TMHMM. Third, we obtained KEGG pathway annotations via the KofamKOALA web server v2020-08-04 (KEGG release 95.0) [11]. A gene was considered to have a full functional annotation (see Suppl. Fig. 4), when it received hits in both the Swissprot and Uniprot databases, was assigned a GO and KEGG annotation, and received at least one assignment to Pfam domains, InterPro domains, PRINTS, PANTHER, SMART, or SignalP. A gene was considered to have no functional annotation when it received not a single hit in any of the above categories (see Suppl. Fig. 4).

We predicted protein targeting for a subset of genes involved in nitrogen metabolism, the pentose phosphate pathway, the Calvin cycle, the TCA cycle, glycine/serine/threonine metabolism, and

glycolysis/gluconeogenesis using a method slightly adapted from published work [12, 13]. More specifically, we used the software programs MitoProt [14], HECTAR v1.3 [15], SignalP-3.0 [16], ASAFind [17], and TargetP-2.0 [18] to predict protein localization to the mitochondria, chloroplasts, or cytoplasm. If a protein had predicted plastid-peptides in HECTAR, SignalP and ASAFind it was classified as plastid-targeted, whereas if it was targeted to the mitochondria by any two of MitoProt, HECTAR, or TargetP it was classified as mitochondria-targeted. As no endoplasmic reticulum-targeted signal peptides were detected, all proteins with no clear targeting were assumed to be active in the cytoplasm. If conflicting results were obtained, proteins were classified as having dual or uncertain targeting.

Homology search in the *S. marinoi* genome

To find orthologs of the *S. marinoi* genes in other diatoms for which a genome is available, we ran OrthoFinder v2.2.6 [19] in DIAMOND mode including nine other diatom proteomes, i.e. *Cyclotella cryptica*, *Cyclotella nana*, *Fistulifera solaris*, *Fragilariopsis cylindrus*, *Nitzschia* sp., *Phaeodactylum tricornutum*, *Pseudonitzschia multiseries*, *Pseudonitzschia multistriata*, and *Seminavis robusta* [13, 20–26]. In addition, we used BLAST+ with an e-value limit of 1e-6 to search for homologs of a series of bacterial genes in the *S. marinoi* genome. This was done for the enzymes *ectA*, *ectB*, and *ectC* which are involved in the ectoine pathway in bacteria. To this end, all available sequences of bacterial enzymes were downloaded from Uniprot, and for each the best hit was retained in BLAST+. At last, we detected all genes that were DE in the experiment of Ferrante et al. [27] on sexual reproduction in *S. marinoi*. Specifically, we performed a BLAST+ search of all genes in Supplementary Tables 6a and 6c of Ferrante et al. [27] against the genome of *S. marinoi*, retaining the best hit.

Differential expression analysis

Using the R-package edgeR v3.34.0 [28], we filtered the gene-level counts to only include genes that have at least one count per million (CPM) in at least three samples. TMM normalization (i.e., weighted trimmed mean of the log expression ratios) was used to eliminate technical variation due to library size and composition [29]. We created a multidimensional scaling (MDS) plot based on Euclidean distances on the gene expression profiles of the pairwise top-500 genes using the R-package limma v.3.48.0 [30]. Subsequently, we used edgeR to fit a quasi-negative binomial generalized linear model (GLMs) [31] for every gene using the glmQLFit function with a group-model design that included each strain-treatment combination. Hypothesis testing was performed using F-tests with the glmQLFTest function. Due to the multiplicity of tests performed for each gene, we used stage-wise testing in stageR v1.14.0 [32]. Specifically, in the screening stage of stageR, we tested the global null hypothesis (i.e., the null hypothesis over all contrasts together) on a 5 % FDR using the Benjamini-Hochberg correction [33]. We then tested all contrasts separately in the confirmation stage, only including genes that were significant in the screening stage, and Holm's method [34] was used to control the within-gene family-wise error rate (FWER) on the adjusted FDR-level of the screening stage, altogether controlling the gene-level FDR at 5 % [32, 35]. Sets of top genes of interest for the average response and individual strains were selected using stageR's FDR-adjusted p value of the global null hypothesis (Padjscreen). In addition, we selected top gene sets for individual contrasts using contrast-specific FDR-controlled p values, as well as logFC values via the Topconfects method [36].

GO enrichment

For each significant gene in the confirmation stage of the stage-wise testing analysis, we extracted Gene Ontology (GO) terms from the InterProScan results and subdivided these into the three main GO categories: Biological Processes (BP), Molecular Function (MF), and Cellular Component (CC). We performed GO enrichment on the results of the individual strains and the average response (first null

hypothesis), and the interaction effects (second null hypothesis). For the individual strains and the average response, we performed GO enrichment for each of the 27 contrasts in CAMERA [37] as implemented in edgeR, separately for each main GO category. In addition, we defined two main categories of genes in the individual strain and average responses, separately for each strain and the average response: (i) upregulated in low salinities, and (ii) downregulated in low salinities. We assigned genes to these two categories based on their expression patterns in the three salinities, visualized using the TMM normalized logarithm of the average expression of each gene in function of salinity, i.e., the log-fitted values of the glmQLFit output (Suppl. Fig. 17). Genes that were up- or downregulated in intermediate salinities were discarded as these sets were too small to perform robust GO enrichment (Suppl. Fig. 17). For each of the two categories, GO enrichment was performed using Fisher's exact test and the *elim* algorithm in the R-package TopGO v2.44.0 [38], separately for each main GO category.

For the interaction effects, genes were assigned to two sets: (i) genes responding in similar directions across strains, and (ii) genes responding in different directions across strains. Set (i) also included genes that were only DE in one strain. To determine to which category a gene belonged, we used the logFC values of DE genes from the contrasts of the individual strains (first hypothesis, Fig. 1C). Specifically, when for a given gene all logFC values of DE contrasts of the strains were positive or negative, a gene belonged to category (i), whereas if both positive and negative logFC values were detected, a gene was assigned to category (ii). Genes that were not DE in any contrast of the individual strains were assigned to (i). Twenty-four genes of (ii) were uniquely DE in one strain but showed both positive and negative logFC values in different contrasts, indicating differences in the direction of DE between different contrasts within a strain, but not across strains. These genes were removed from (ii) and added to (i) prior to GO enrichment, because they represent a difference in magnitude of the response across strains. This ensured (ii) only included genes that differed in the direction of DE across strains, whereas (i) included all other genes with interaction effects which exhibited differences in magnitude, but not direction, across different strains. When assigning genes, we did not distinguish between the three different sets of salinity contrasts (8–16, 16–24, and 8–24) because this would have reduced the set of DE genes too much to allow for robust GO enrichment. Subsequently, we performed GO enrichment of the two sets of interaction-effect genes in TopGO as outlined above. For all TopGO analyses, the selected set of genes was compared to the full set of genes in the genome of *S. marinoi*. For both the TopGO and CAMERA results, we used REVIGO [39] to summarize significantly ($p < 0.05$) enriched GO terms, using a 0.5 similarity cut-off value and the SimRel score as similarity measure [40], following earlier work on diatoms [41].

SUPPLEMENTARY PROTOCOL

This section outlines the recipe for the artificial sea water (ASW medium) used in our experiment.

We used ddH_2O to create ASW medium, and added nutrients, vitamins, and salts. First, Suppl. Table 1 gives an overview of the stock solutions of each nutrient used in our ASW medium. Per liter of ASW medium, 1 mL of each stock solution listed in Suppl. Table 1 was added. In addition, we added 0.5 mL of the vitamin stock of standard L1 medium to each liter ASW medium.

Suppl. Table 1. Stock solutions of the nutrients used in ASW medium. Dilutions were done in ddH_2O.

Nutrient	Stock (g/250mL)
$NaHCO_3$	3.1504
H_3BO_3	5.9975
K_2HPO_4	2.1772
$NaNO_3$	10.6238
KNO_3	12.6375
$NASiO_3 . 9H_2O$	14.2100
NH_4Cl	6.6498
$FeCl_3 . 6H_2O$	0.1250
$FeSO_4 . 7H_2O$	0.1286
$Na_2EDTA . 2H_2O$	1.0887
$MnCl_2 . 4H_2O$	0.0445

In addition to the nutrients listed in Suppl. Table 1, we also added a mix of trace nutrients to the medium (Suppl. Table 2). Primary stocks of all trace nutrients were combined into a single secondary stock solution, by combining 250 µL of each primary stock and adding up to a total of 250 mL ddH_2O. Per liter of ASW medium, 1 mL of the secondary trace nutrient stock was used.

Suppl. Table 2. Primary stock solutions of the trace nutrients used to create a single secondary stock solution for ASW medium. Dilutions were done in ddH_2O.

Nutrient	Primary stock (g/250mL)
$CuSO_4 . 5H_2O$	0.1249
$CuCl_2 . 2H_2O$	0.0852
$ZnSO_4 . 7H_2O$	2.8760
$ZnCl_2$	1.3630
$CoCl_2 . 6H_2O$	2.9741
$Na_2MoO_4 . 2H_2O$	5.4439
Na_2SeO_3	0.0043
Na_3VO_4	0.4598

To obtain ASW medium with different salinities but equal nutrient concentrations, we created two stock solutions of medium at two different salinities (0 and 30). Suppl. Table 3 gives the quantities of salt needed to obtain the desired salinities.

Suppl. Table 3. Salts (in grams per liter medium) added to the ASW medium to obtain a salinity of 0 or 30.

	Desired salinity	
**Salt**	**0**	**30**
NaCl	0.000	24.582
MgSO4 . 7H2O	0.044	5.110
MgCl2 . 6H2O	0.000	4.271
KCl	0.024	0.732
CaCl2 . 2H2O	0.048	1.465
**total salts (g)**	0.116	36.160

Finally, to obtain the salinities used in our experiment (8, 16, and 24), the media at salinity 0 and 30 were mixed at different proportions (Suppl. Table 4), thus varying the salt concentrations while keeping the nutrients constant.

Suppl. Table 4. Proportions of media at salinities 0 and 30 needed to obtain the experimental salinities.

Desired salinity	0	30
8	733 mL	267 mL
16	467 mL	533 mL
24	200 mL	800 mL

SUPPLEMENTARY FIGURES

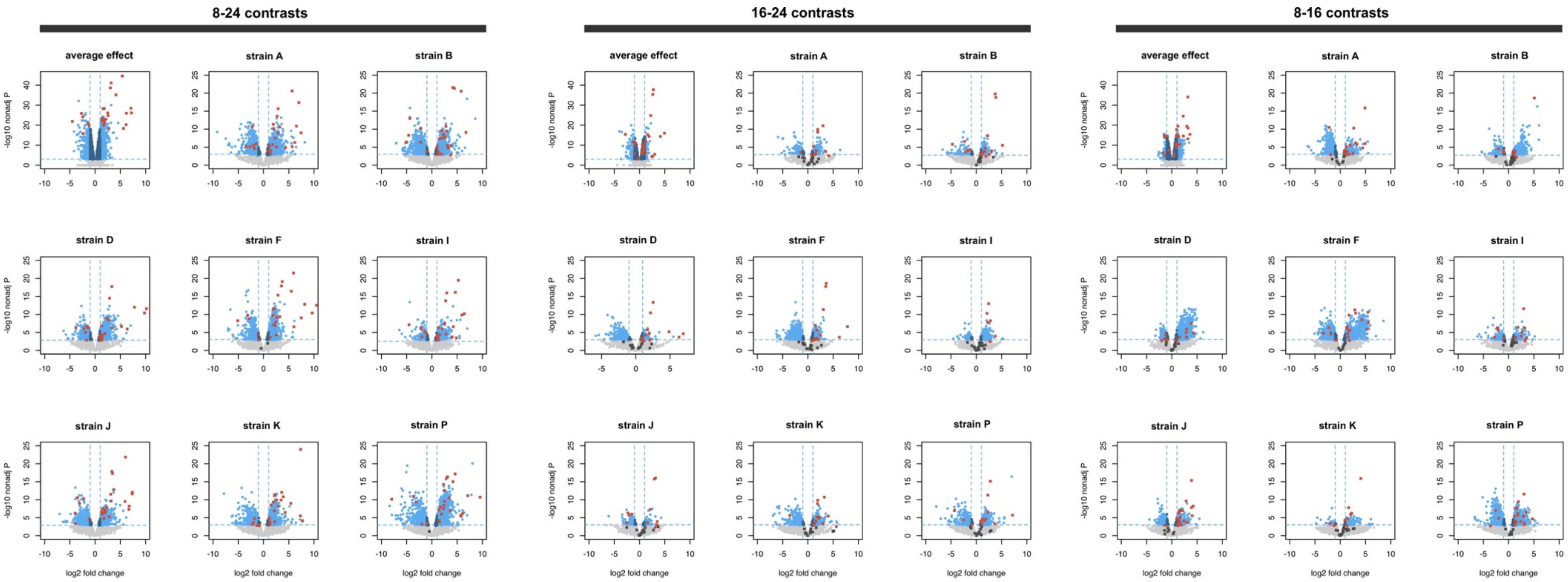

Suppl. Fig. 1. Volcano plots of all the contrasts for the average response and individual strains. The plots depict the logFC versus the log10 5 % FDR-adjusted *p* values. All values above the horizontal dotted blue lines are significant, and all values below this line are not significant. DE core response genes are indicated with red squares. Dark grey squares indicate core response genes that are not significant in a given contrast.

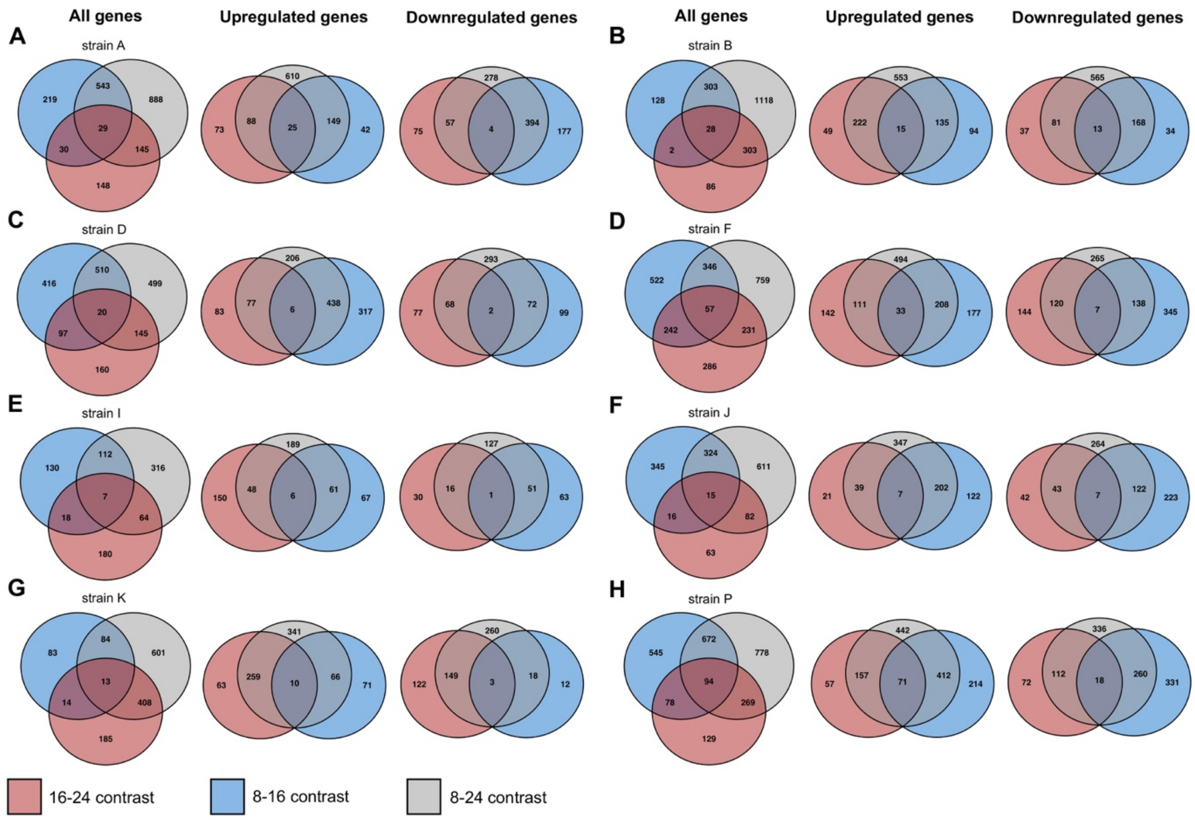

Suppl. Fig. 2. Venn diagrams showing the number of unique and shared DE genes between the three salinity contrasts for each strain. a Strain A. **b** Strain B. **c** Strain D. **d** Strain F. **e** Strain I. **f** Strain J. **g** Strain K. **h** Strain P. For each strain, we distinguished between the full set of DE genes (‘All genes’), and genes that are up- or downregulated. Note that some DE genes showed an opposite expression pattern in different contrasts (upregulation in one contrast, but downregulation in another contrast). These genes were removed from the Venn diagrams showing up- and downregulated genes only: these equaled 30, 2, 109, 259, 18, 17, 14 and 83 genes for strains A, B, D, F, I, J, K and P, respectively. Most of these DE genes were shared only between the 16–24 and 8–16 contrasts and were not DE in the 8–24 contrast.

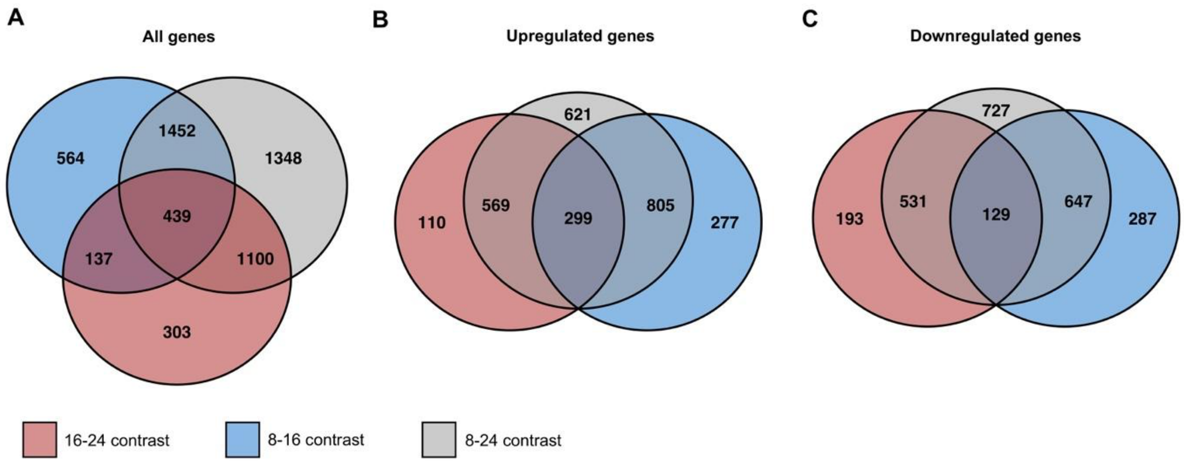

Suppl. Fig. 3. Venn diagrams showing the number of unique and shared DE genes between the three salinity contrasts for the average response. a All the genes DE in the average response. **b** Genes upregulated in the average response. **c** Genes downregulated in the average response. Note that 148 DE genes in **a** showed an opposite expression pattern in different contrasts (upregulation in one contrast, but downregulation in another contrast). These genes were removed from **b** and **c**. Most of these DE genes were shared only between the 16–24 and 8–16 contrasts and were not DE in the 8–24 contrast.

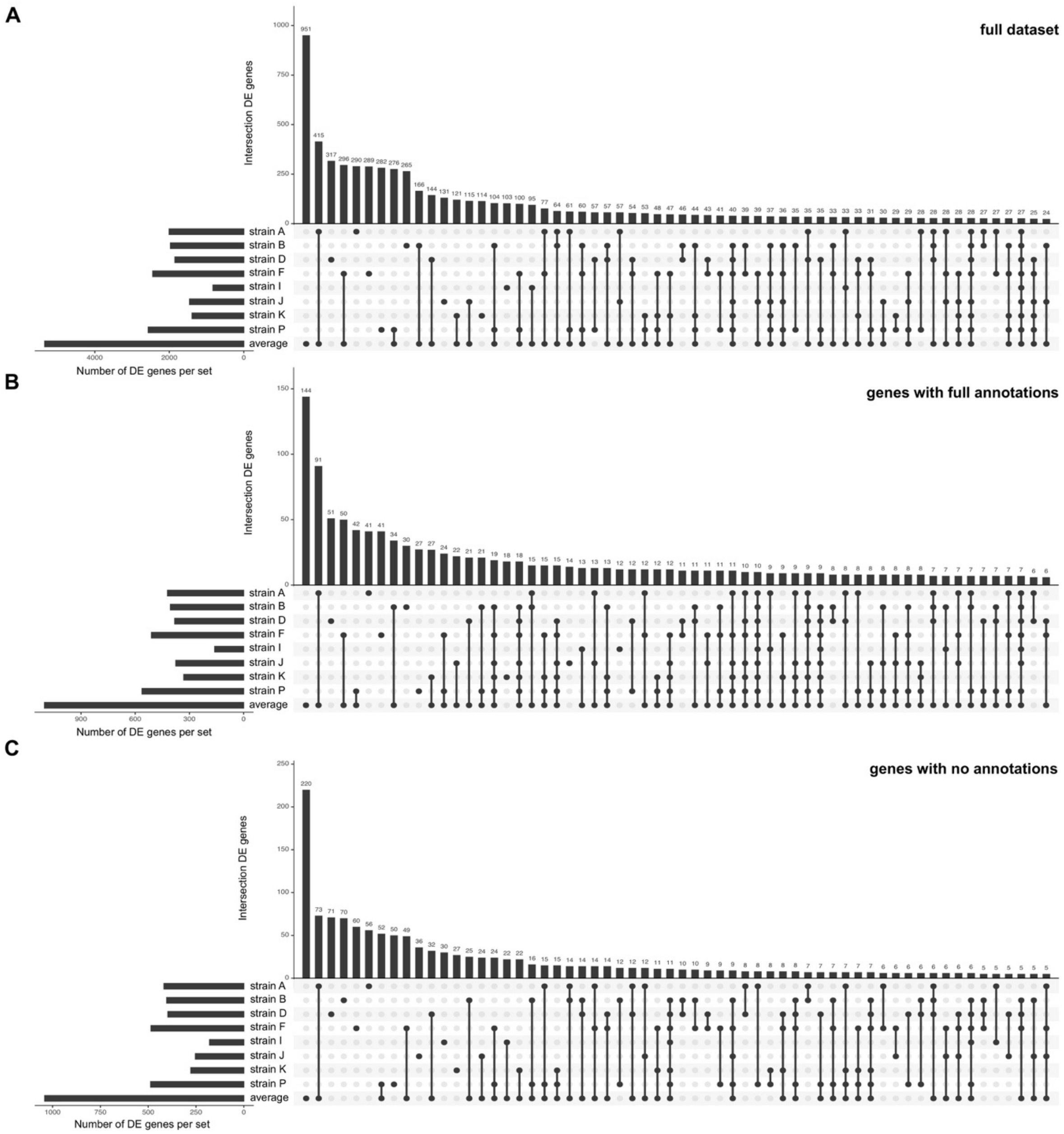

Suppl. Fig. 4. Upset plots showing uniquely DE and shared DE genes between strains, combining data of all three salinity contrasts together (8–24, 8–16, 16–24). a Full dataset. **b** Genes with full annotation, i.e., with assigned GO terms, KEGG annotations, Uniprot and Swissprot annotations, and at least one assignment to the InterPro, PANTHER, Pfam, SMART, PRINTS, or SignalP databases as determined by InterProScan. **c** Genes with no annotations in the categories of set (**b**). For all upset plots, information from all salinity contrasts was combined.

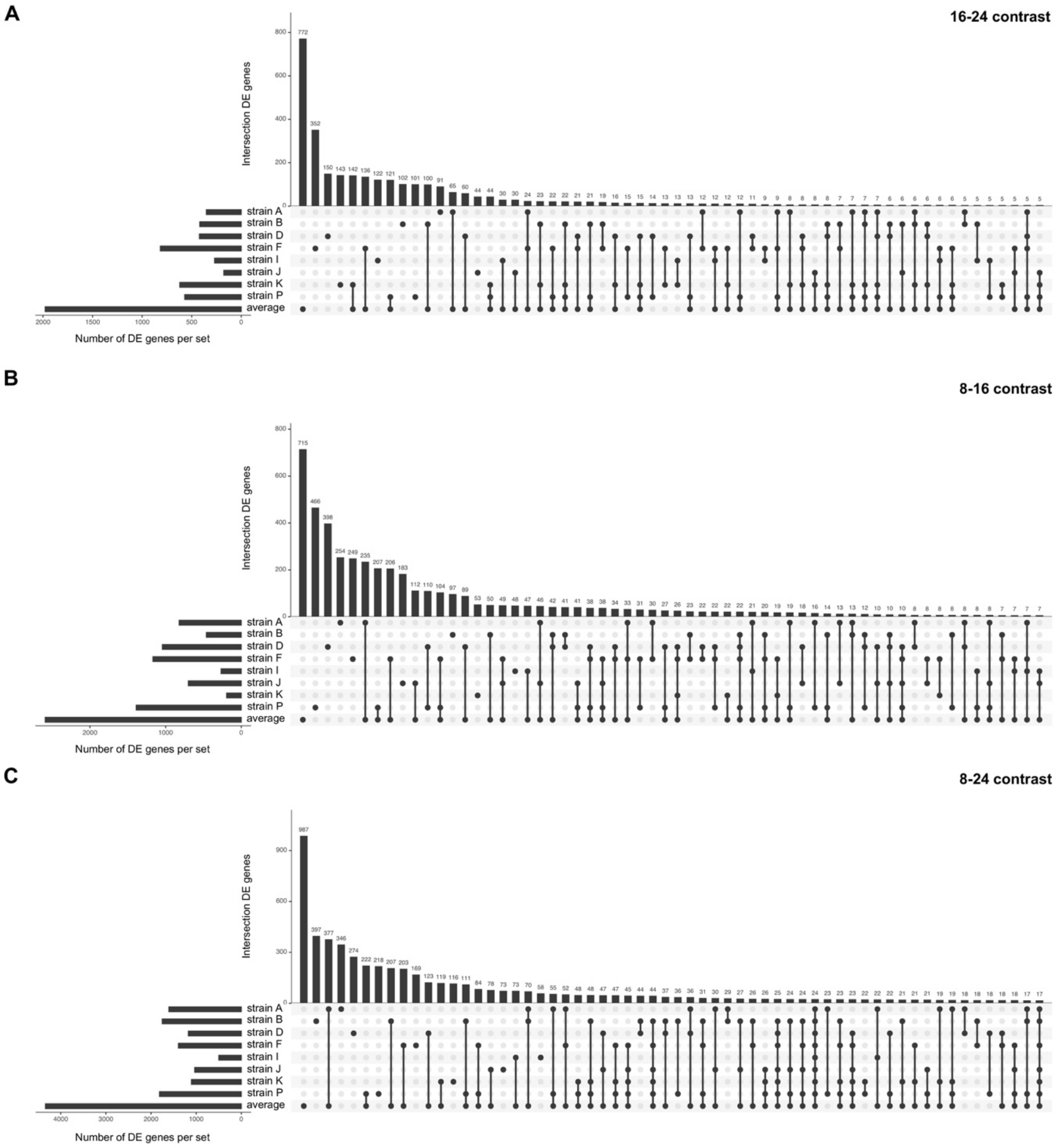

Suppl. Fig. 5. Upset plots showing unique and shared DE genes between strains, separately for each salinity contrast. a 16–24 contrast. **b** 8–16 contrast. **c** 8–24 contrast.

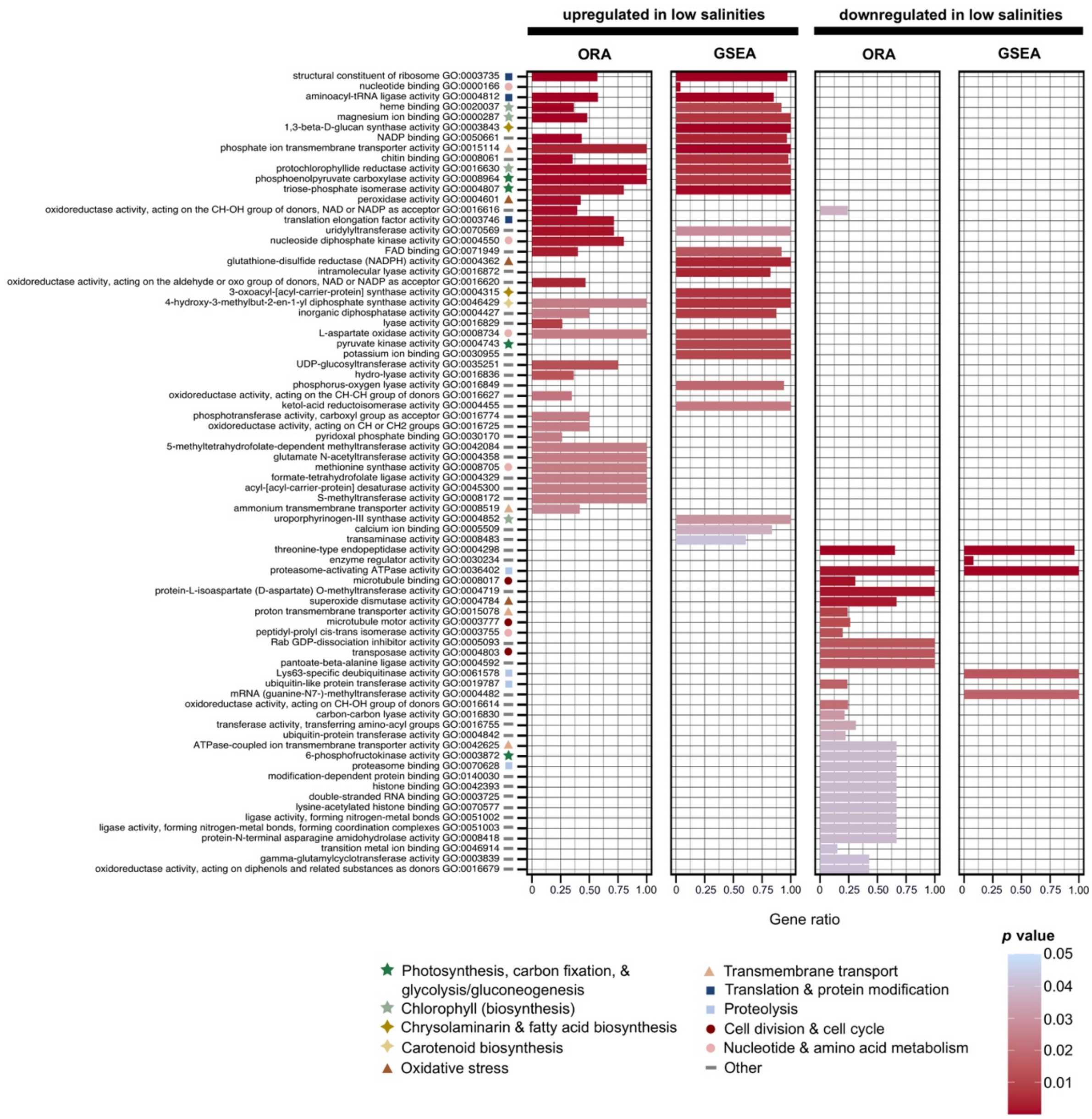

Suppl. Fig. 6. GO enrichment on the average response of *S. marinoi* to low salinities: Molecular Function. The results of two types of GO enrichment analyses are shown: ORA (in topGO) and GSEA (in CAMERA), after removal of redundant terms by REVIGO. For ORA, we classified the total set of DE genes in the average response in two categories, distinguishing between genes that are up- or downregulated in low salinities, regardless of salinity contrast (see Supplementary Methods for more details). For CAMERA, we performed GSEA analyses on each individual contrast separately, showing only the 8–24 contrast in this figure. Barplot height indicates the proportion of genes that are DE with a given GO-term to the total number of genes with this GO-term in the genome of *S. marinoi*. The barplots are colored according to *p* value. Within the set of up- and downregulated genes, the GO-terms are ranked from lowest to highest *p* value, using the lowest of two *p* values from ORA or GSEA. Symbols indicate major categories of cellular processes to which a GO-term belongs. Only Molecular Function GO-terms are shown.

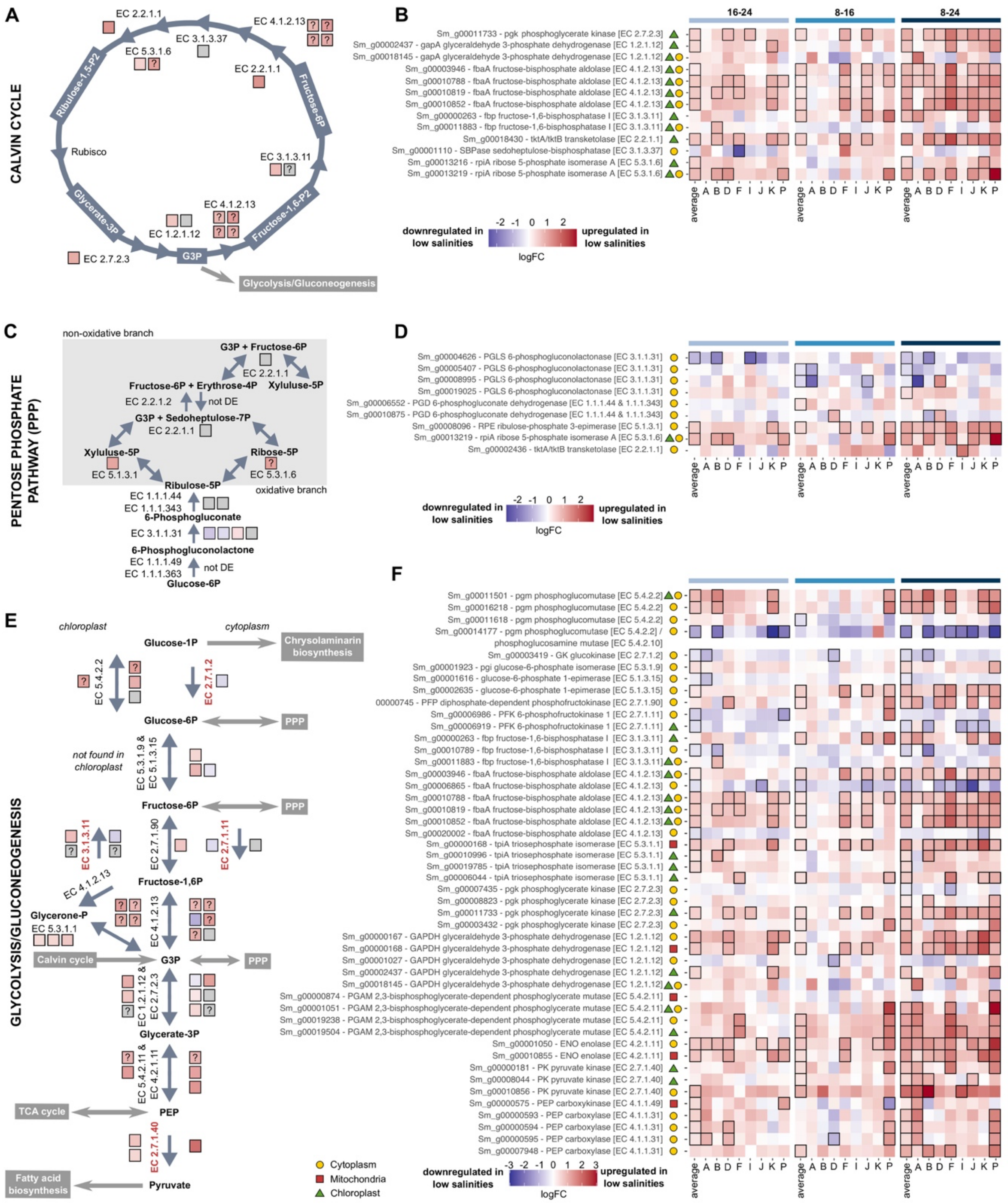

Suppl. Fig. 7. Gene expression of genes involved in carbohydrate metabolism, showing pathways and heatmaps. a, b Calvin cycle. **c, d** Pentose phosphate pathway. **e, f** Glycolysis/gluconeogenesis. All heatmaps (**b, d, f**) show logFC values of the 16–24, 8–16 and 8–24 contrasts of the average response and the eight strains. All visualized genes are DE in at least one contrast. Significant contrasts are outlined in black. Symbols next to the gene names in the heatmaps denote protein targeting. In case protein targeting was unclear, multiple symbols are used. Each colored square in the pathway figures (**a, c, e**) corresponds with a single gene, and is colored according to the logFC values of the 8–24 contrast of the average response. Squares with question marks indicate proteins with unclear targeting. In panel (**e**), the genes left and right of the pathway arrows are targeted to the chloroplast and cytoplasm, respectively. When protein targeting is unclear, a gene is shown on both sides. Irreversible steps are shown in red. In panel (**f**), gene *Sm_g00010788* is DE in all 8–24 contrasts, except for strain I which appears DE due to colored edge lines from neighboring squares. Similarly for the 8–24 contrast, genes *Sm_g00006865*, *Sm_g00010855,* and *Sm_g00010856* are not DE in strains F, A, and A, respectively.

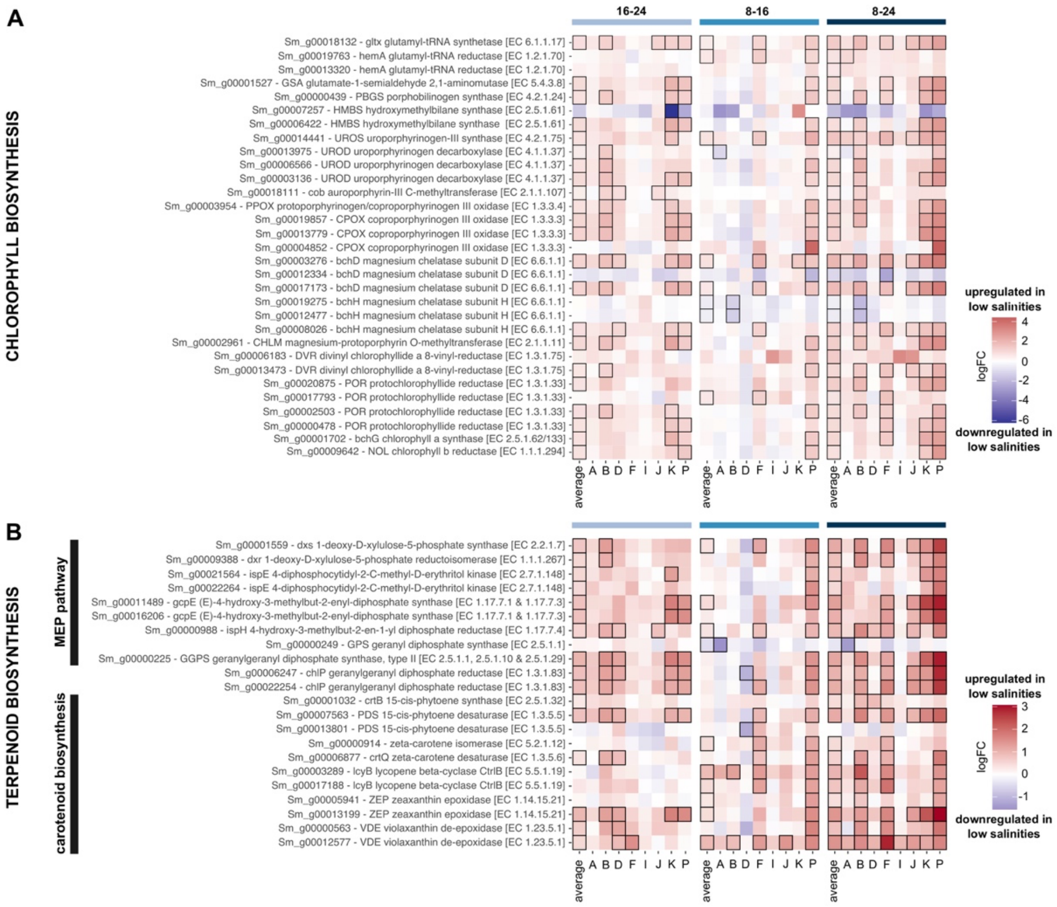

Suppl. Fig. 8. Gene expression of genes involved in chlorophyll and terpenoid biosynthesis. a Chlorophyll-biosynthesis genes. **b** Terpenoid-biosynthesis genes. Both the non-mevalonate (MEP) pathway and the downstream pathway to the biosynthesis of carotenoids are shown. The mevalonate pathway is not shown, as only a few genes of this pathway were DE. The heatmaps show logFC values of the 16–24, 8–16 and 8–24 contrasts of the average response and the eight strains. All visualized genes are DE in at least one contrast. Contrasts that were significant are outlined in black.

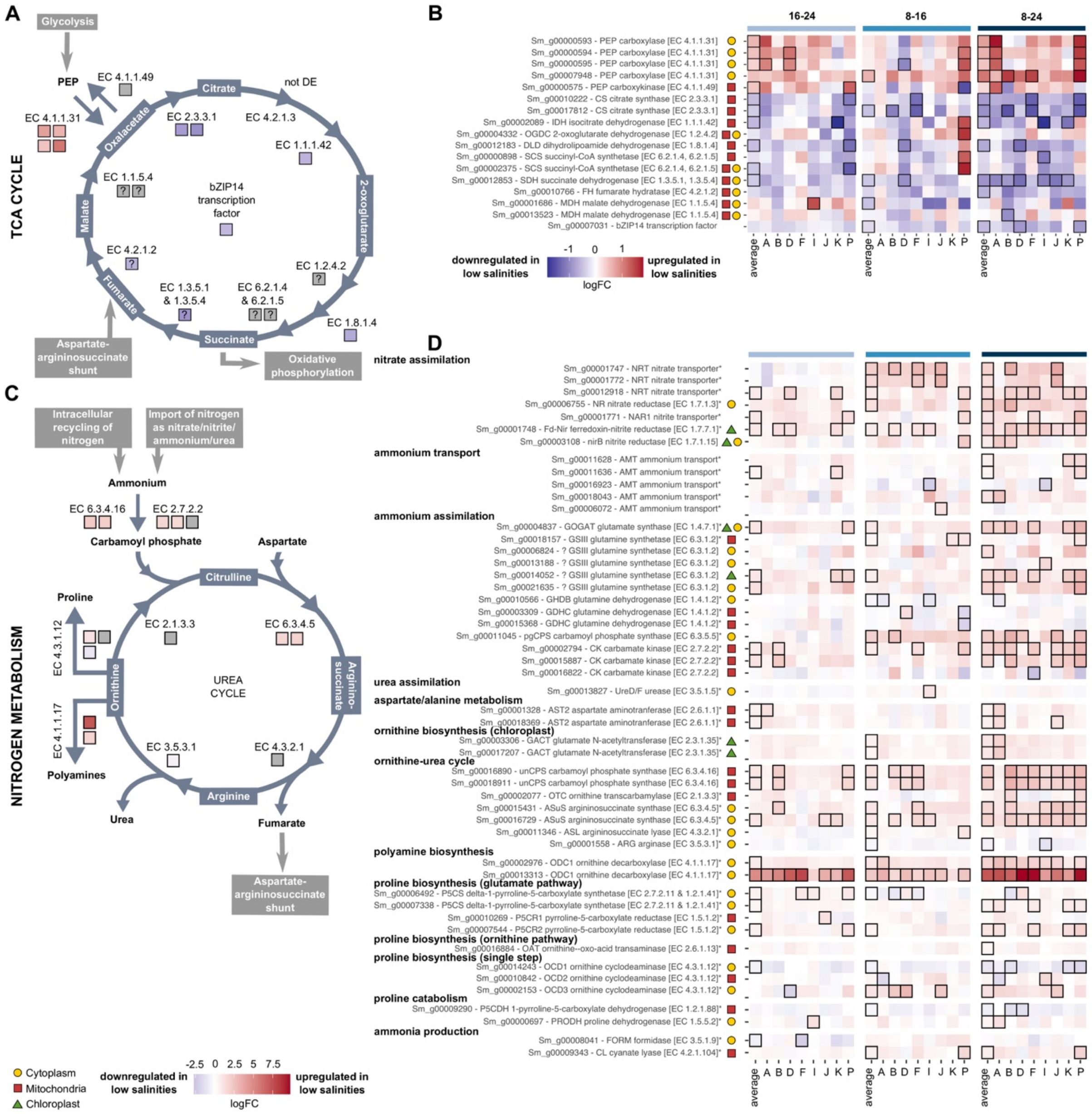

Suppl. Fig. 9. Gene expression of genes involved in the TCA cycle and nitrogen metabolism, showing pathways and heatmaps. a, b TCA cycle. **c, d** Nitrogen metabolism, based on [42]. All heatmaps (**b, d**) show logFC values of the 16–24, 8–16 and 8–24 contrasts of the average response and the eight strains. All visualized genes are DE in at least one contrast. Contrasts that were significant are outlined in black. Symbols next to the gene names in the heatmaps denote protein targeting. In case the protein targeting was unclear, multiple symbols are used. Each colored square in the pathway figures (**a, c**) corresponds with a single gene, and is colored according to the logFC values of the 8–24 contrast of the average response. Squares filled with a question mark indicate proteins with unclear targeting. In panel (**b**), gene *Sm_g00007948* is not DE in the 8–24 contrast of strain A, but appears DE in the figure due to colored edge lines from neighboring squares.

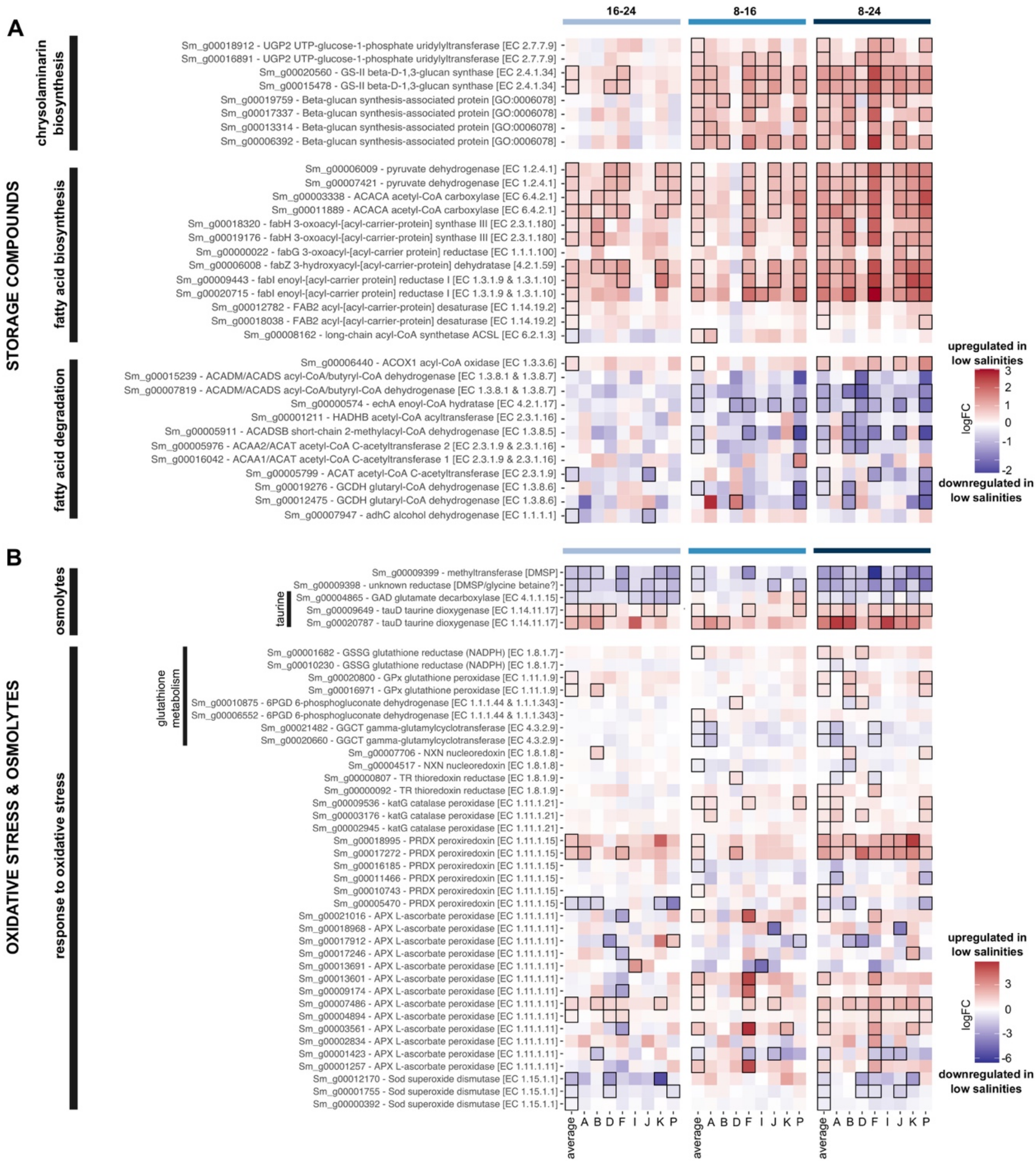

Suppl. Fig. 10. Gene expression of genes involved in biosynthesis of storage compounds, osmolyte function, and response to oxidative stress. a Biosynthesis of chrysolaminarin (β-1,3-glucans and β-1,6-glucans), and biosynthesis and degradation of fatty acids. **b** Osmolytes and oxidative stress. Proline and the xanthophyll cycle genes are not shown in (**b**), but are incorporated in the nitrogen metabolism (Suppl. Fig. 9) and terpenoid biosynthesis (Suppl. Fig. 8), respectively. The heatmaps show logFC values of the 16–24, 8–16 and 8–24 contrasts of the average response and the eight strains. All visualized genes are DE in at least one contrast. Contrasts that were significant are outlined in black. In panel (**a**), gene *Sm_g00020560* (chrysolaminarin biosynthesis) is DE in all 8–24 contrasts, except for strain D which appears DE in the figure due to colored edge lines from neighboring squares. Similarly for the 8–24 contrast, genes *Sm_g00011889*, *Sm_g00020560, Sm_g00007421, Sm_g00003338* and *Sm_g00000022* are not DE for strains D, D, A and K, respectively. Same for gene *Sm_g00011889* in the 16–24 contrast (strain B). In panel (**b**), gene *Sm_g00009398* is not DE in the 16–24 and 8–24 contrasts of strain K. Similarly, the 8–24 contrast of gene *Sm_g00004865* is not DE for strains A and J, and the 8–24 contrast of gene *Sm_g00009649* is not DE for strain I.

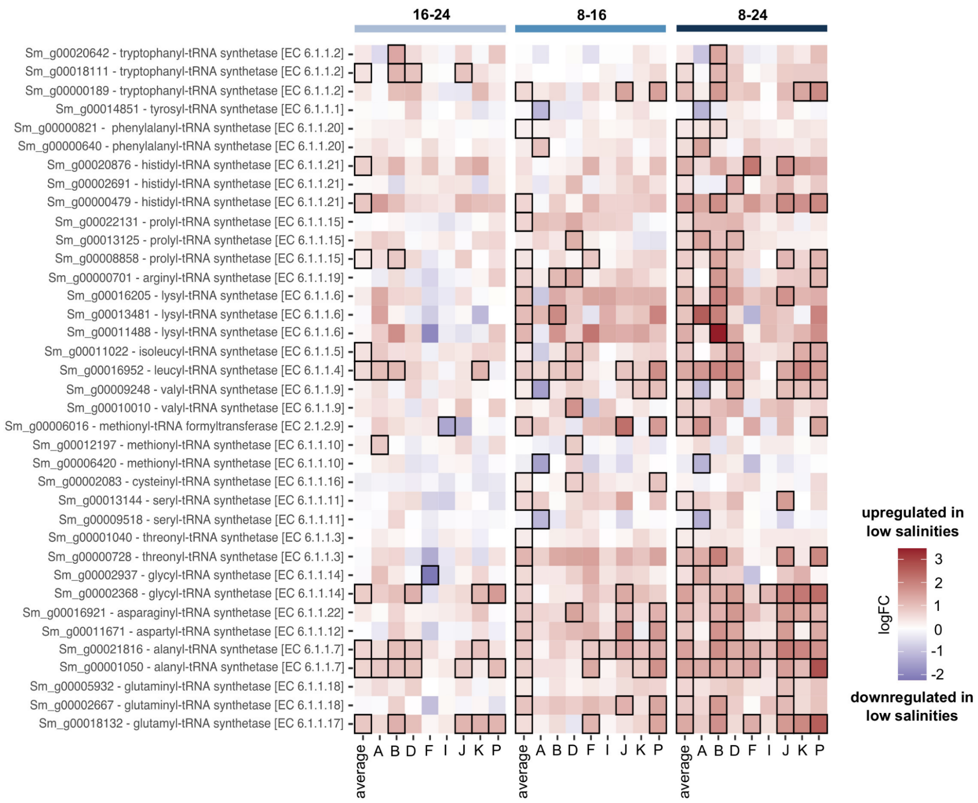

Suppl. Fig. 11. Gene expression of genes involved in tRNA-aminoacylation. The heatmap shows logFC values of the 16–24, 8–16 and 8–24 contrasts of the average response and the eight strains. All visualized genes are DE in at least one contrast. Contrasts that were significant are outlined in black. For the 8–24 contrast, genes *Sm_g00000821* and *Sm_g00011671* are not DE for strains A and K, respectively, but appear DE in the figure due to colored edge lines from neighboring squares.

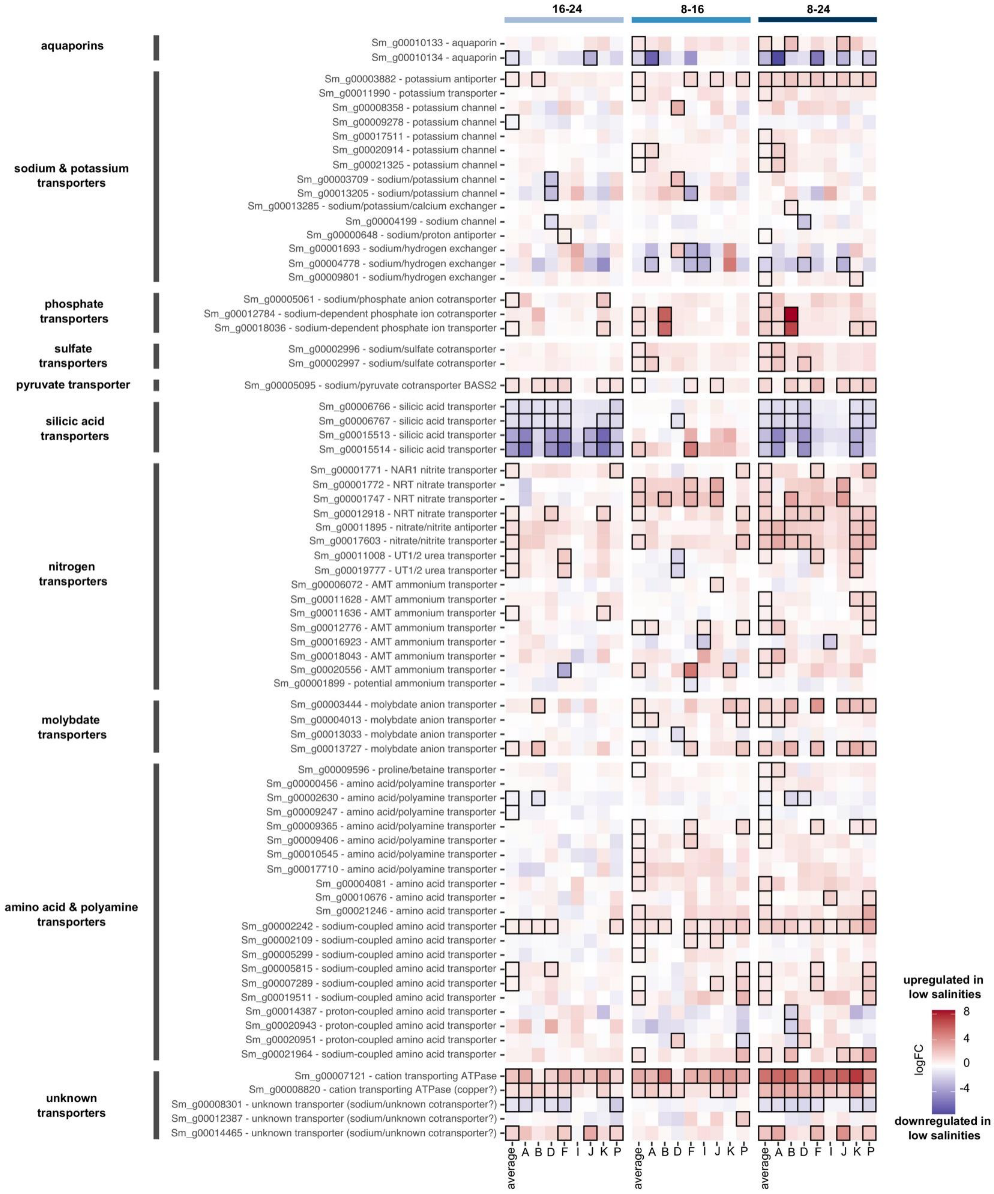

Suppl. Fig. 12. Gene expression of transmembrane transporter genes. The heatmap shows logFC values of the 16–24, 8–16 and 8–24 contrasts of the average response and the eight strains. All visualized genes are DE in at least one contrast. Contrasts that were significant are outlined in black.

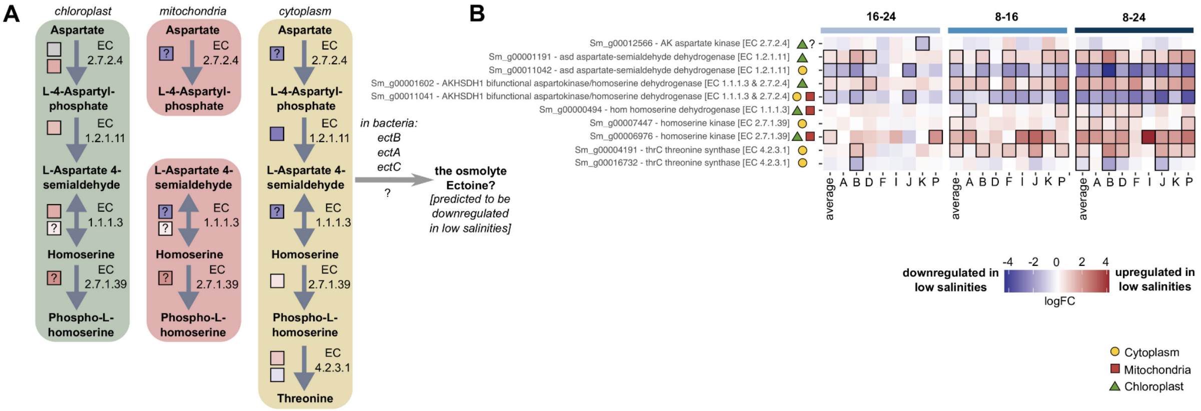

Suppl. Fig. 13. Gene expression of genes involved in the first steps of the glycine/serine/threonine pathway, showing the pathway and heatmap. a Visualization of the first steps of the pathway, with distinction between the chloroplast, mitochondria, and cytoplasm. **b** Heatmaps showing logFC values of the 16–24, 8–16 and 8–24 contrasts of the average response and the eight strains. All visualized genes are DE in at least one contrast. Contrasts that were significant are outlined in black. Symbols next to the gene names in the heatmaps denote protein targeting. In case the protein targeting was unclear, multiple symbols are used. Each colored square in the pathway figure (**a**) corresponds with a single gene and is colored according to the logFC values of the 8–24 contrast of the average response. Squares filled with a question mark indicate proteins with unclear targeting. In case protein targeting was unclear, a gene is shown in multiple cell compartments. The grey arrow in (**a**) shows the presumed location of the branch towards ectoine biosynthesis. In panel (**b**), gene *Sm_g00001602* is not DE in the 8-16 and 8-24 contrasts of strain I, but appears DE in the figure due to colored edge lines from neighboring squares. Similarly for the 8–24 contrast of genes *Sm_g00000494* (strains B and D) and *Sm_g00007447* (strain B).

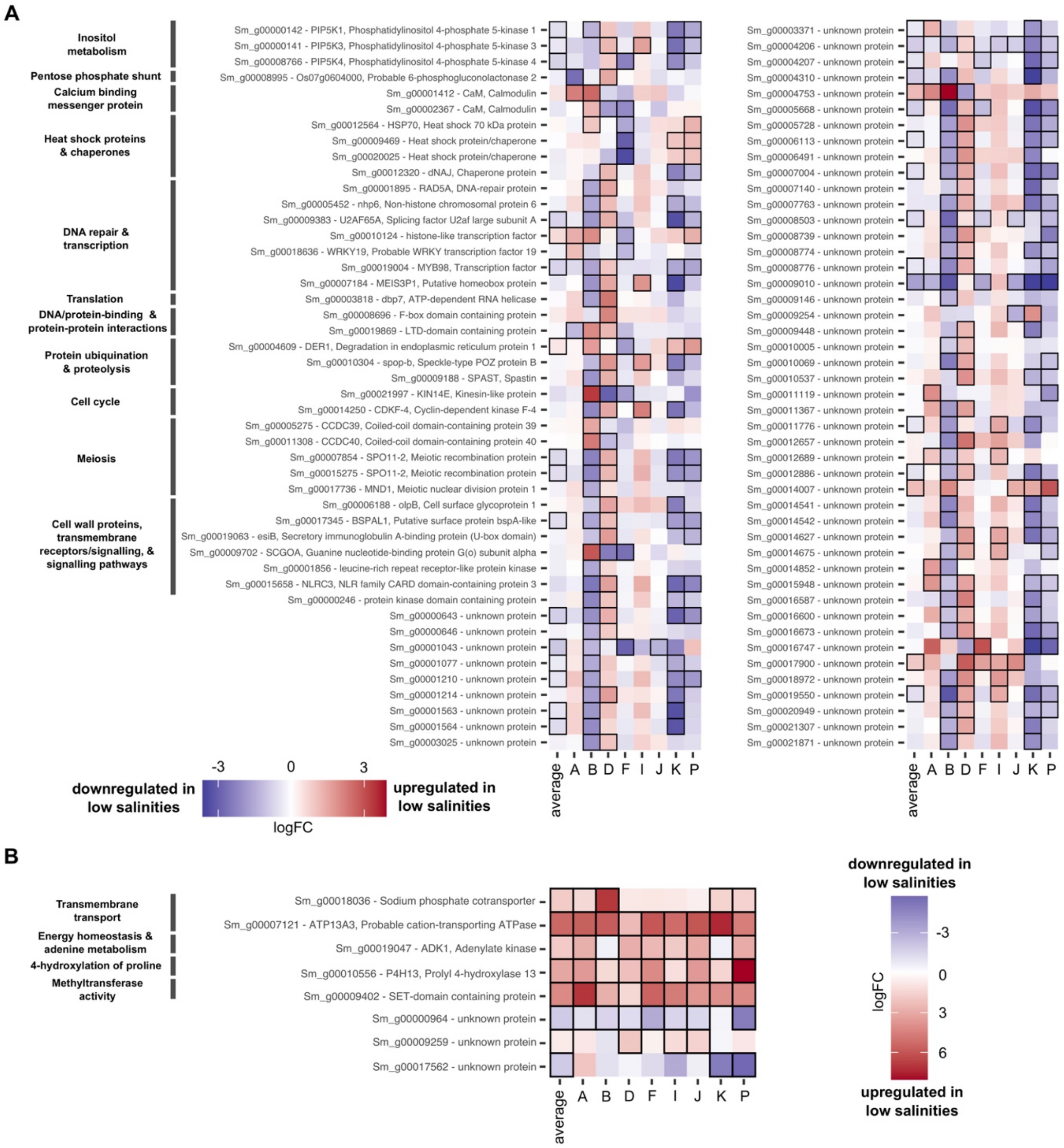

Suppl. Fig. 14. Gene expression of the top-100 genes with significant interaction effects between strains. Heatmap showing logFC values of the 8–24 contrasts of the average response and the eight strains. All visualized genes belong to the top-100 genes identified in the interaction-effect test and were selected using stageR's FDR-adjusted *p* value of the global null hypothesis (Padjscreen). When possible, functional annotations are indicated. Sixty-one of the top genes lacked (clear) functional annotations. **a** Genes which are DE in different directions in different strains. This set includes 92 genes. Note that some of the genes in the figure only show significant logFC values that are either positive or negative: these genes are classified as genes with DE in different directions because of significant logFC values in the 8–16 or 16–24 contrasts which are not shown in the figure (see Supplementary Methods for more information). **b** Genes which are DE in the same direction in different strains, but to a significantly different magnitude. This set includes 8 genes. Only contrasts significant in the individual strains were taken into account to classify genes into (**a**) or (**b**). Contrasts that were significant are outlined in black. In panel (**a**), genes *Sm_g00004609* and *Sm_g00008503* are not DE in the 8–24 contrast of strain D, but appear DE in the figure due to colored edge lines from neighboring squares. Similarly, in panel (**b**) for gene *Sm_g00019047* (strains B and K).

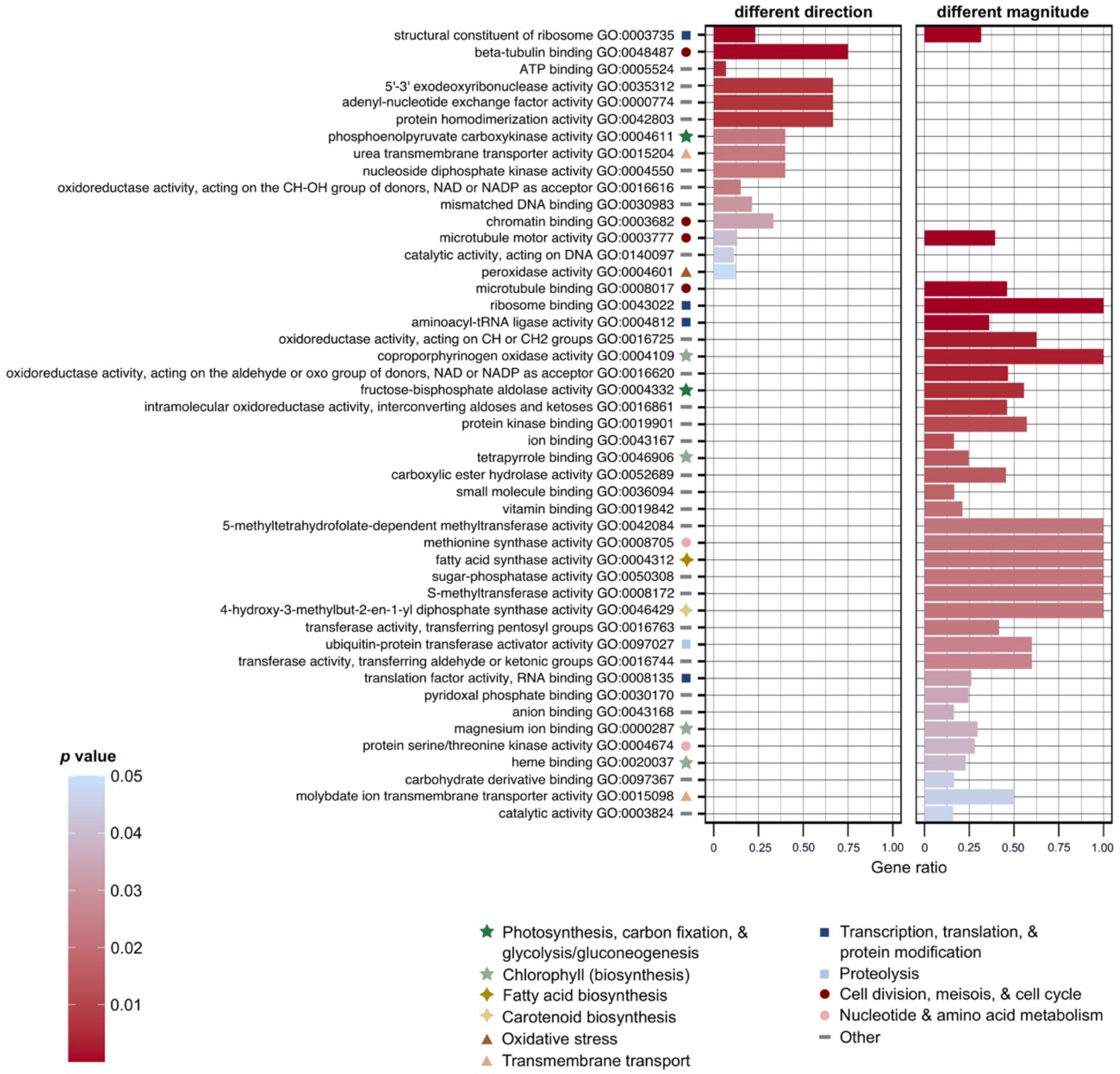

Suppl. Fig. 15. GO enrichment on the interaction effects: Molecular Function. The barplots visualize the significant GO terms retrieved by ORA (topGO, Fisher's exact test, *elim* algorithm) after removal of redundant GO terms by REVIGO. Two sets of GO enrichment were carried out which distinguished between genes that differ significantly between strains in the direction or magnitude of their response to low salinities. Barplot height indicates the proportion of genes that are DE with a given GO term to the total number of genes with this GO term in the genome of *S. marinoi*. The barplots are colored, and the GO terms ranked, according to *p* value. Symbols indicate major categories of cellular processes to which a GO term belongs. Only Molecular Function GO terms are shown.

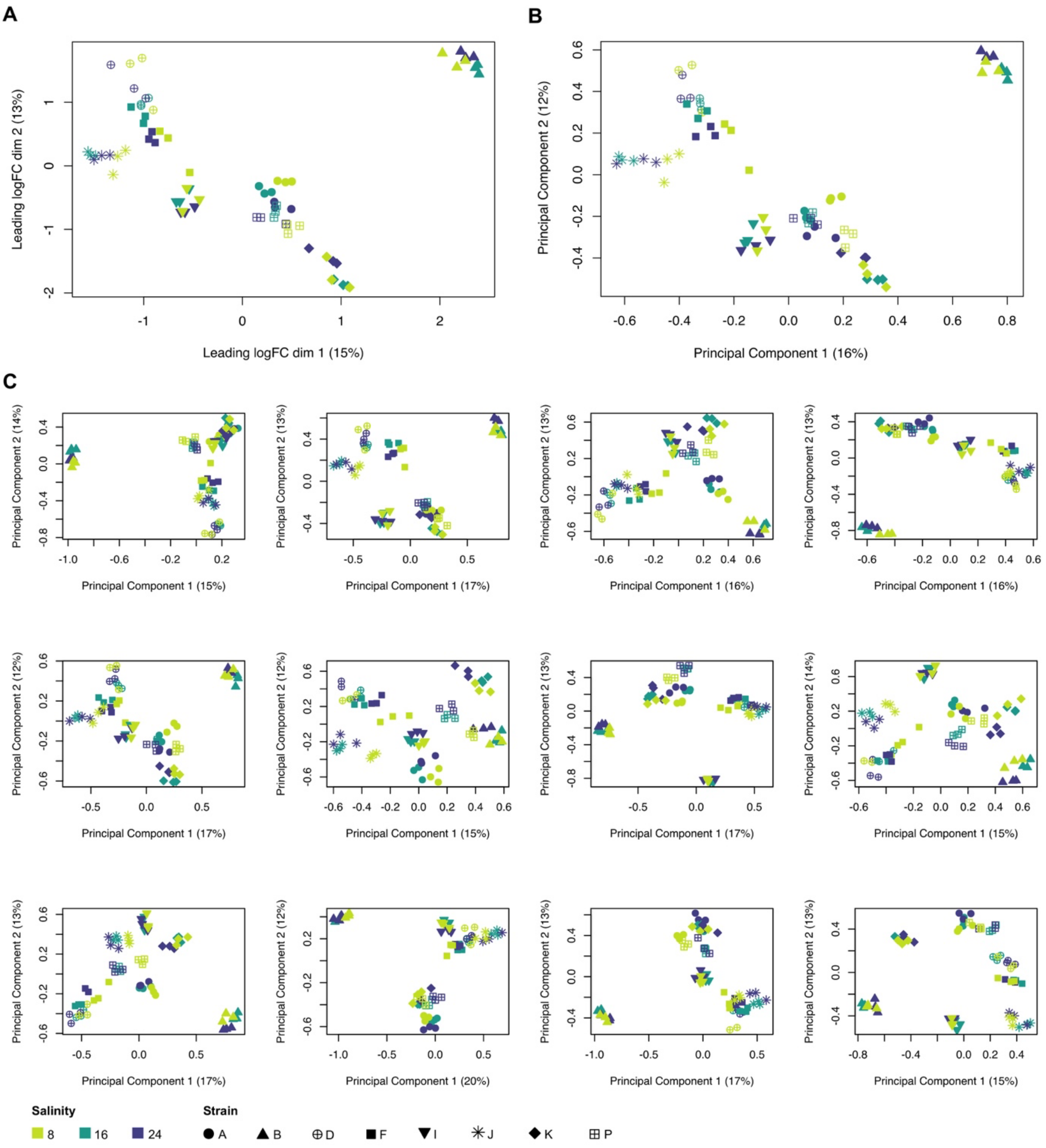

Suppl. Fig. 16. Intraspecific variation in the response of Baltic *S. marinoi* to low salinities, upon removal of genes involved in the cell cycle, mitosis, and meiosis. Genes previously identified as playing a role in sexual reproduction in *S. marinoi* by [27], as well as genes with GO terms related to cell cycle, mitosis, and meiosis, were removed prior to making the plots. In addition, only genes that were clearly expressed were retained (a CPM of 3 in at least 9 samples). A total of 12 705 genes were retained for the plots. **a** Multidimensional scaling (MDS, PCoA) plot. Distances between the samples are based on logFC changes in the top-500 genes, selecting the top-500 genes separately for each pairwise comparison between the samples (*gene.selection* = *'pairwise'*). **b** Multidimensional scaling (MDS, PCA) plot. Distances between the samples are based on logFC changes in the total of 12 705 genes (*gene.selection* = *'common'*). **c** Multidimensional scaling (MDS, PCA) plots. Each plot shows a randomly chosen set of 500 genes. Distances between the samples are based on logFC changes in the 500 selected genes (*gene.selection* = *'common'*).

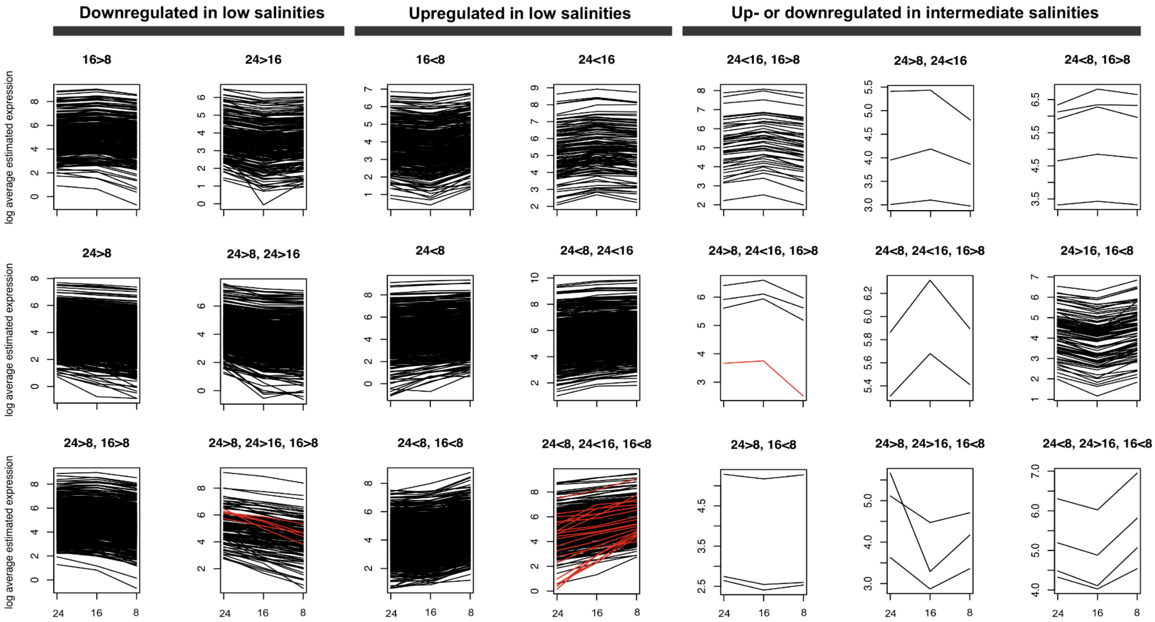

Suppl. Fig. 17. TMM normalized logarithm of the average expression of each DE gene in function of salinity. Gene expression data were averaged over all eight strains in this study using the log-fitted values of the glmQLFit output. Genes are assigned to different plots based on the DE contrasts and the direction of their response (e.g., genes in the 16>8 category are only DE in the 8–16 contrast and are always downregulated in salinity 8 relative to 16). Plots are ranked based on the overall direction of their response: downregulated in low salinities, upregulated in low salinities, and up- or downregulated in intermediate salinities. These first two sets were used as input for the ORA (in topGO) analysis on GO enrichment. Expression-curves indicated in red represent the 27 core-response genes.

SUPPLEMENTARY REFERENCES

1. Scholin CA, Herzog M, Sogin M, Anderson DM. Identification of group- and strain-specific genetic markers for globally distributed *Alexandrium* (Dinophyceae). II. Sequence analysis of a fragment of the LSU rDNA gene. *J Phycol* 1994; **30**: 999–1011.
2. Guillard RRL, Lorenzen CJ. Yellow-green algae with chlorophyllide c 1, 2. *J Phycol* 1972; **8**: 10–14.
3. Guillard RRL, Hargraves PE. *Stichochrysis immobilis* is a diatom, not a chrysophyte. *Phycologia* 1993; **32**: 234–236.
4. Wood AM, Everroad RC, Wingard LM. Chapter 18. Measuring growth rates in microalgal cultures. In: Andersen RA (ed). *Algal Culturing Techniques*. 2005. Elsevier Academic Press, pp 269–288.
5. Andrew S. FastQC: a quality control tool for high throughput sequence data. Available online at: http://www.bioinformatics.babraham.ac.uk/projects/fastqc. 2010.
6. Sun K. Ktrim: an extra-fast and accurate adapter- and quality-trimmer for sequencing data. *Bioinformatics* 2020; **36**: 3561–3562.
7. Dobin A, Gingeras TR. Mapping RNA-seq Reads with STAR. *Curr Protoc Bioinformatics* 2015; **51**: 11.14.1-11.14.19.
8. Anders S, Pyl PT, Huber W. HTSeq--a Python framework to work with high-throughput sequencing data. *Bioinformatics* 2015; **31**: 166–169.
9. Altschul SF, Gish W, Miller W, Myers EW, Lipman DJ. Basic local alignment search tool. *J Mol Biol* 1990; **215**: 403–410.
10. Jones P, Binns D, Chang H-Y, Fraser M, Li W, McAnulla C, et al. InterProScan 5: genome-scale protein function classification. *Bioinformatics* 2014; **30**: 1236–1240.
11. Aramaki T, Blanc-Mathieu R, Endo H, Ohkubo K, Kanehisa M, Goto S, et al. KofamKOALA: KEGG Ortholog assignment based on profile HMM and adaptive score threshold. *Bioinformatics* 2020; **36**: 2251–2252.
12. Traller JC, Cokus SJ, Lopez DA, Gaidarenko O, Smith SR, McCrow JP, et al. Genome and methylome of the oleaginous diatom *Cyclotella cryptica* reveal genetic flexibility toward a high lipid phenotype. *Biotechnol Biofuels* 2016; **9**: 258.
13. Onyshchenko A, Roberts WR, Ruck EC, Lewis JA, Alverson AJ. The genome of a nonphotosynthetic diatom provides insights into the metabolic shift to heterotrophy and constraints on the loss of photosynthesis. *New Phytol* 2021; **232**: 1750–1764.
14. Claros MG. MitoProt, a Macintosh application for studying mitochondrial proteins. *Comput Appl Biosci* 1995; **11**: 441–447.
15. Gschloessl B, Guermeur Y, Cock JM. HECTAR: a method to predict subcellular targeting in heterokonts. *BMC Bioinformatics* 2008; **9**: 393.
16. Bendtsen JD, Nielsen H, von Heijne G, Brunak S. Improved prediction of signal peptides: SignalP 3.0. *J Mol Biol* 2004; **340**: 783–795.
17. Gruber A, Rocap G, Kroth PG, Armbrust EV, Mock T. Plastid proteome prediction for diatoms and other algae with secondary plastids of the red lineage. *Plant J* 2015; **81**: 519–528.
18. Almagro Armenteros JJ, Salvatore M, Emanuelsson O, Winther O, von Heijne G, Elofsson A, et al. Detecting sequence signals in targeting peptides using deep learning. *Life Sci Alliance* 2019; **2**: e201900429.
19. Emms DM, Kelly S. OrthoFinder: phylogenetic orthology inference for comparative genomics. *Genome Biol* 2019; **20**: 238.
20. Osuna-Cruz CM, Bilcke G, Vancaester E, De Decker S, Bones AM, Winge P, et al. The *Seminavis robusta* genome provides insights into the evolutionary adaptations of benthic diatoms. *Nat Commun* 2020; **11**: 3320.
21. Basu S, Patil S, Mapleson D, Russo MT, Vitale L, Fevola C, et al. Finding a partner in the ocean: molecular and evolutionary bases of the response to sexual cues in a planktonic diatom. *New Phytol* 2017; **215**: 140–156.
22. Bowler C, Allen AE, Badger JH, Grimwood J, Jabbari K, Kuo A, et al. The *Phaeodactylum* genome reveals the evolutionary history of diatom genomes. *Nature* 2008; **456**: 239–244.

23. Mock T, Otillar RP, Strauss J, McMullan M, Paajanen P, Schmutz J, et al. Evolutionary genomics of the cold-adapted diatom *Fragilariopsis cylindrus*. *Nature* 2017; **541**: 536–540.
24. Tanaka T, Maeda Y, Veluchamy A, Tanaka M, Abida H, Maréchal E, et al. Oil accumulation by the oleaginous diatom *Fistulifera solaris* as revealed by the genome and transcriptome. *Plant Cell* 2015; **27**: 162–176.
25. Armbrust EV, Berges JA, Bowler C, Green BR, Martinez D, Putnam NH, et al. The genome of the diatom *Thalassiosira pseudonana*: ecology, evolution, and metabolism. *Science* 2004; **306**: 79–86.
26. Roberts WR, Downey KM, Ruck EC, Traller JC, Alverson AJ. Improved reference genome for *Cyclotella cryptica* CCMP332, a model for cell wall morphogenesis, salinity adaptation, and lipid production in diatoms (Bacillariophyta). *G3* 2020; **10**: 2965–2974.
27. Ferrante MI, Entrambasaguas L, Johansson M, Töpel M, Kremp A, Montresor M, et al. Exploring molecular signs of sex in the marine diatom *Skeletonema marinoi*. *Genes* 2019; **10**.
28. Robinson MD, McCarthy DJ, Smyth GK. edgeR: a Bioconductor package for differential expression analysis of digital gene expression data. *Bioinformatics* 2010; **26**: 139–140.
29. Robinson MD, Oshlack A. A scaling normalization method for differential expression analysis of RNA-seq data. *Genome Biol* 2010; **11**: R25.
30. Ritchie ME, Phipson B, Wu D, Hu Y, Law CW, Shi W, et al. limma powers differential expression analyses for RNA-sequencing and microarray studies. *Nucleic Acids Res* 2015; **43**: e47.
31. Lund SP, Nettleton D, McCarthy DJ, Smyth GK. Detecting differential expression in RNA-sequence data using quasi-likelihood with shrunken dispersion estimates. *Stat Appl Genet Mol Biol* 2012; **11**.
32. Van den Berge K, Soneson C, Robinson MD, Clement L. stageR: a general stage-wise method for controlling the gene-level false discovery rate in differential expression and differential transcript usage. *Genome Biol* 2017; **18**: 151.
33. Benjamini Y, Hochberg Y. Controlling the false discovery rate: a practical and powerful approach to multiple testing. *J R Stat Soc Series B Stat Methodol* 1995; **57**: 289–300.
34. Holm S. A Simple Sequentially rejective multiple test procedure. *Scand Stat Theory Appl* 1979; **6**: 65–70.
35. Heller R, Manduchi E, Grant GR, Ewens WJ. A flexible two-stage procedure for identifying gene sets that are differentially expressed. *Bioinformatics* 2009; **25**: 1019–1025.
36. Harrison PF, Pattison AD, Powell DR, Beilharz TH. Topconfects: a package for confident effect sizes in differential expression analysis provides a more biologically useful ranked gene list. *Genome Biol* 2019; **20**: 67.
37. Wu D, Smyth GK. Camera: a competitive gene set test accounting for inter-gene correlation. *Nucleic Acids Res* 2012; **40**: e133.
38. Alexa A, Rahnenführer J. Gene set enrichment analysis with topGO. *Bioconductor Improv* 2009; **27**.
39. Supek F, Bošnjak M, Škunca N, Šmuc T. REVIGO summarizes and visualizes long lists of gene ontology terms. *PLoS One* 2011; **6**: e21800.
40. Schlicker A, Domingues FS, Rahnenführer J, Lengauer T. A new measure for functional similarity of gene products based on Gene Ontology. *BMC Bioinformatics* 2006; **7**: 302.
41. Bilcke G, Van den Berge K, De Decker S, Bonneure E, Poulsen N, Bulankova P, et al. Mating type specific transcriptomic response to sex inducing pheromone in the pennate diatom *Seminavis robusta*. *ISME J* 2021; **15**: 562–576.
42. Smith SR, Dupont CL, McCarthy JK, Broddrick JT, Oborník M, Horák A, et al. Evolution and regulation of nitrogen flux through compartmentalized metabolic networks in a marine diatom. *Nat Commun* 2019; **10**: 4552.
